# SPATS2, negatively regulated by miR-145-5p, promotes hepatocellular carcinoma progression through regulating cell cycle

**DOI:** 10.1038/s41419-020-03039-y

**Published:** 2020-10-09

**Authors:** Gang Dong, Shanshan Zhang, Shen Shen, Lulu Sun, Xuemei Wang, Haiyu Wang, Jie Wu, Tingting Liu, Chaoyan Wang, Huanbin Wang, Taiying Lu, Benchen Rao, Zhigang Ren

**Affiliations:** 1 Department of Ultrasound, the First Affiliated Hospital of Zhengzhou University, 450052 Zhengzhou, China; 2 Department of Infectious Diseases, the First Affiliated Hospital of Zhengzhou University, 450052 Zhengzhou, China; 3 Gene Hospital of Henan Province; Precision Medicine Center, the First Affiliated Hospital of Zhengzhou University, 450052 Zhengzhou, China; 4 Department of Magnetic Resonance Imaging, the First Affiliated Hospital of Zhengzhou University, 450052 Zhengzhou, China; 5 Department of Ultrasound, The Central Hospital of Xuchang City, 461000 Xuchang, China; 6 Department of Oncology, the First Affiliated Hospital of Zhengzhou University, 450052 Zhengzhou, China

**Keywords:** Oncogenes, Cell growth, Prognostic markers

## Abstract

Spermatogenesis associated serine rich 2 (SPATS2) has been reported to contribute to the tumorigenesis of multiple malignancies. The molecular function of SPATS2 in hepatocellular carcinoma (HCC) is still not fully understood. In this study, we aimed to investigate the expression pattern and function roles of SPATS2 in HCC. The regulation of SPATS2 expression was also explored. We found that SPATS2 was highly expressed in HCC tissues in comparison with that in adjacent normal tissues. High expression of SPATS2 was associated with vascular invasion, advanced TNM stages, tumor multiplicity, and poor survival. Functionally, SPATS2 was found to promote the proliferation and metastasis of HCC cells both in vitro and in vivo, while knockdown of SPATS2 enhanced apoptosis and G1 arrest of HCC cells in vitro. Mechanistically, bioinformatics analysis revealed that MiR-145-5p directly targeted SPATS2 and functional rescue experiments verified that MiR-145-5p overexpression could abolish the effect of SPATS2 on the regulation of HCC malignant phenotype. Taken together, our findings suggest that SPATS2 functions as an oncogene in HCC. The MiR-145-5p/SPATS2 axis provides a novel mechanism underlying HCC progression and may serve as a potential therapeutic target for HCC.

## Introduction

Hepatocellular carcinoma (HCC) is the sixth most common neoplasm and the third leading cause of cancer-related deaths worldwide^1,2^. Although great progress has been made in treatment strategies, such as surgical resection, trans-arterial chemoembolization, radiofrequency ablation, and liver transplantation, the five-year survival rate of HCC is less than 10%^3^. The poor prognosis of HCC is mainly due to the diagnosis at late stage and its recurrence potential. Thus, it is of great importance to further explore the mechanisms of HCC progression and identify novel HCC diagnostic markers.

The spermatogenesis associated serine rich 2 (SPATS2), which is predicted as a cytoplasmic RNA-binding protein, is initially discovered in testis and plays an important role in spermatogenesis^4^. SPATS2 has been found significantly downregulated in human oral epithelial cells after treatment with bisphenol A^5^. Subsequently, SPATS2 was identified as a novel diagnostic biomarker in squamous cell carcinoma^6^. SPATS2 participated in the process of lncRNA SNHG5-mediated colorectal cancer cell survival^7^. In addition, SPATS2 was associated with prostate cancer progression^8^. Recently, Xing et al. reported that SPATS2 was highly expressed in liver cancer and has the high diagnostic ability^9^. However, the expression profile and function of SPATS2 in HCC remains unclear.

MiRNAs are a class of small non-coding RNAs that post-transcriptionally regulate gene expression through targeting their 3′-untranslated regions (3-UTRs)^10,11^. Emerging evidence has widely confirmed the critical role of miRNAs in tumorigenesis. MiRNAs could promote carcinogenesis through targeting tumor suppressor genes or inhibit tumor development by suppressing oncogenes^12–16^. MiR-145-5p has been characterized as a tumor suppressor in gastric cancer^17^, colorectal cancer^18^, breast cancer^19^, and bladder cancer^20^. Nevertheless, study has shown that MiR-145-5p could promote lung cancer development, which indicates that MiR-145-5p might have different biological functions in different cancer types^21^. Additionally, MiR-145-5p expression was significant decreased in hepatitis B virus-associated HCC and low miR-145-5p expression predicted poor prognosis of HCC patients^22–24^. Nevertheless, how MiR-145-5p regulates the progression of HCC is not fully understood.

In this study, we found that SPATS2 expression was upregulated in HCC tissues. High expression of SPATS2 was associated with adverse clinicopathological features and worse outcome of HCC patients. SPATS2 knockdown significantly suppressed HCC cell growth and invasion, while promoted apoptosis and G1 arrest of HCC cells in vitro. Furthermore, SPATS2 knockdown suppressed HCC tumor growth and metastasis in vivo. Mechanistically, we identified that MiR-145-5p directly targeted SPATS2 and overexpression of miR-145-5p inhibited HCC cell growth and invasion. Our data suggest that the MiR-145-5p/SPATS2 axis exerts vital function in HCC development and might be a promising therapeutic target for HCC.

## Results

### SPATS2 is upregulated in HCC tissues and associated with poor prognosis of HCC patients

**Fig. 1:**
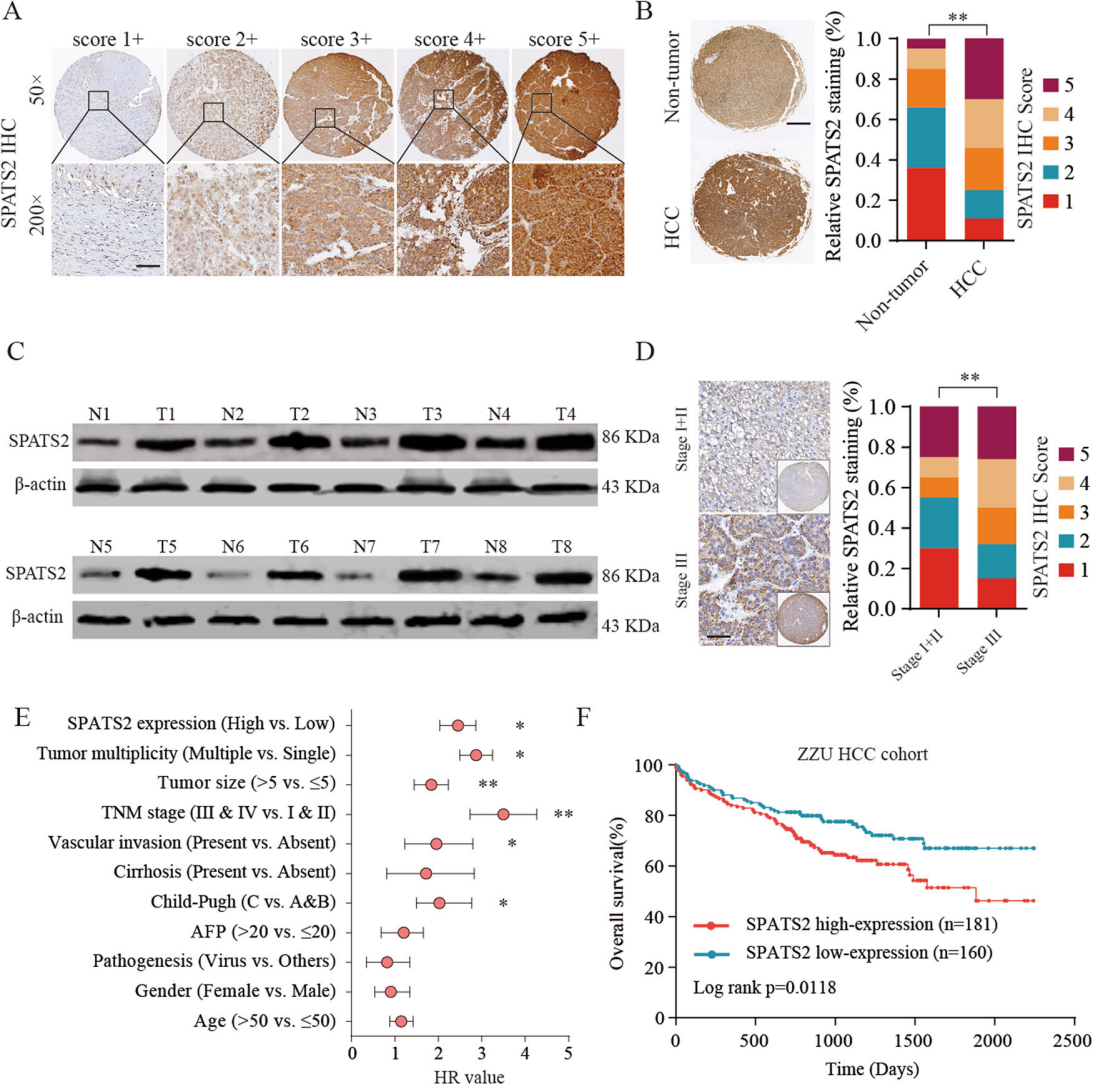
SPATS2 is upregulated in HCC tissues and associated with poor prognosis of HCC patients. **a** Representative SPATS2 immunohistochemical (IHC) staining patterns with different staining scores in HCC tissues. **b** Representative SPATS2 IHC staining in HCC tissues and adjacent non-tumor tissues (left panel) and quantification of IHC staining score distribution between HCC tissues and adjacent non-tumor tissues (right panel). **c** The expression of SPATS2 protein in 8-paired HCC tissues and adjacent non-tumor tissues was analyzed by western blot. **d** Representative SPATS2 IHC staining and quantification of IHC staining score distribution in HCC tissues with different TNM stages. **e** Univariate analysis of the association between SPATS2 expression and different clinicopathological features. AFP, alpha-fetoprotein. **f** Kaplan–Meier analysis of overall survival (OS) in HCC patients with high or low SPATS2 expression in ZZU HCC cohort. Chi-square test was used to compare the levels of SPATS2 expression and various clinicopathological parameters of HCC patients. Survival curves calculation and survival curve plotting used the Kaplan–Meier method. *p < 0.05, **p < 0.01.

To determine the expression profile of SPATS2 in most common cancers, Pan-cancer analysis was performed based on TCGA and GTEX databases. We found SPATS2 mRNA expression was frequently upregulated in most cancers (Supplementary Fig. 1). To investigate the potential role of SPATS2 in HCC, we first examined SPATS2 expression in paired HCC tissues and adjacent normal tissues by IHC staining and western blot. Representative IHC staining of HCC tissues with different scores was shown in Fig. 1a. IHC staining showed that SPATS2 was significantly enhanced in HCC tissues in comparison with matched nonneoplastic counterparts (Fig. 1b). Consistent with the IHC staining results, western blot results of SPATS2 expression in 8-paired tissues also showed that the protein levels of SPATS2 were markedly higher in HCC tissues (Fig. 1c). Furthermore, Stage III HCC tissues had significantly higher SPATS2 staining scores compared with that in Stage I + II HCC tissues (*p* < 0.01, Fig. 1d). We also analyzed the association between SPATS2 expression and clinic-pathological characteristics of HCC (Table 1). Moreover, Univariate Cox regression analysis showed that high SPATS2 expression was a potential independent risk indicator for survival in HCC patients (Fig. 1e). Kaplan–Meier analysis revealed that HCC patients with high SPATS2 expression had significantly shorter survival times than that in patients with low expression of SPTAS2 (Fig. 1f).

**Table 1:** Correlation of clinic-pathological features with SPATS2 expression in ZZU HCC TMA cohort.

Clinic-pathological features	Sum	SPATS2 expression		*P*-value
		Low expression (*n* = 160)	High expression (*n* = 181)	
Age (years)				
≤53	172	92	80	0.927
>53	169	68	101	
Gender				
Male	265	130	135	0.140
Female	76	30	46	
Pathogenesis				
Virus	288	133	155	0.523
Others	53	27	26	
Cirrhosis				
Present	315	150	165	0.368
Absent	26	10	16	
Vascular invasion				
Absent	302	153	149	**0.001**
Present	39	7	32	
TNM stage				
Stage I and II	267	137	130	**0.002**
Stage III and IV	74	23	51	
Tumor size(cm)				
≤5	190	100	90	**0.017**
>5	151	60	91	
AFP				
≤20	166	86	80	0.078
>20	175	71	101	
Tumor multiplicity				
Single	261	131	130	**0.028**
Multiple	80	29	51	

Bold values indicate statistical significance, *P* < 0.05.

To further investigate whether SPATS2 could be a key biomarker in predicting clinical outcome of HCC patients with surgery, univariate and multivariate analysis were performed. The univariate analysis identified that Child–Pugh (*p* = 0.029), vascular invasion (*p* = 0.003), TNM stage (*p* = 0.000), tumor size (*p* = 0.004), tumor multiplicity (*p* = 0.019), and SPATS2 expression (*p* = 0.033) were significantly associated with survival. These factors were then included in the multivariate analysis and the result demonstrated that SPATS2 expression (*p* = 0.029) along with vascular invasion (*p* = 0.039), TNM stage (*p* = 0.001) and tumor multiplicity (*p* = 0.041) were independent prognostic prediction factors for HCC patients who received surgery (Table 2).

**Table 2:** Correlation of clinic-pathological features with SPATS2 expression in ZZU HCC cohort.

	Univariate analysis			Multivariate analysis		
	HR	95% CI	*P*-value	HR	95% CI	*P*-value
Age (>53 vs. ≤53)	1.143	0.884–1.413	0.863			
Gender (Female vs. ≤ Male)	0.846	0.534–1.342	0.479			
Pathogenesis (Virus vs. ≤Others)	0.784	0.341–1.341	0.243			
AFP (>20 vs. ≤20)	1.270	0.684–1.654	0.347			
Cirrhosis (Present vs. Absent)	1.515	0.812–2.825	0.192			
Vascular invasion (Present vs. Absent)	1.848	1.225–2.793	**0.003**	2.126	1.884–2.985	**0.019**
TNM stage (III & IV vs. I & II)	3.333	2.262–4.902	**0.000**	3.954	3.047–5.178	**0.000**
Tumor size (>5 vs. ≤5)	1.751	1.198–2.564	**0.004**	1.499	0.926–1.887	0.274
Tumor multiplicity (Multiple vs. Single)	2.964	2.176–3.47	**0.019**	1.984	1.698–2.659	**0.041**
SPATS2 expression (High vs. Low)	3.265	2.560–4.165	**0.005**	2.731	2.014–3.654	**0.029**

### Bioinformatics analysis of the expression and prognostic value of SPATS2 in TCGA HCC cohort and GEO database

**Fig. 2:**
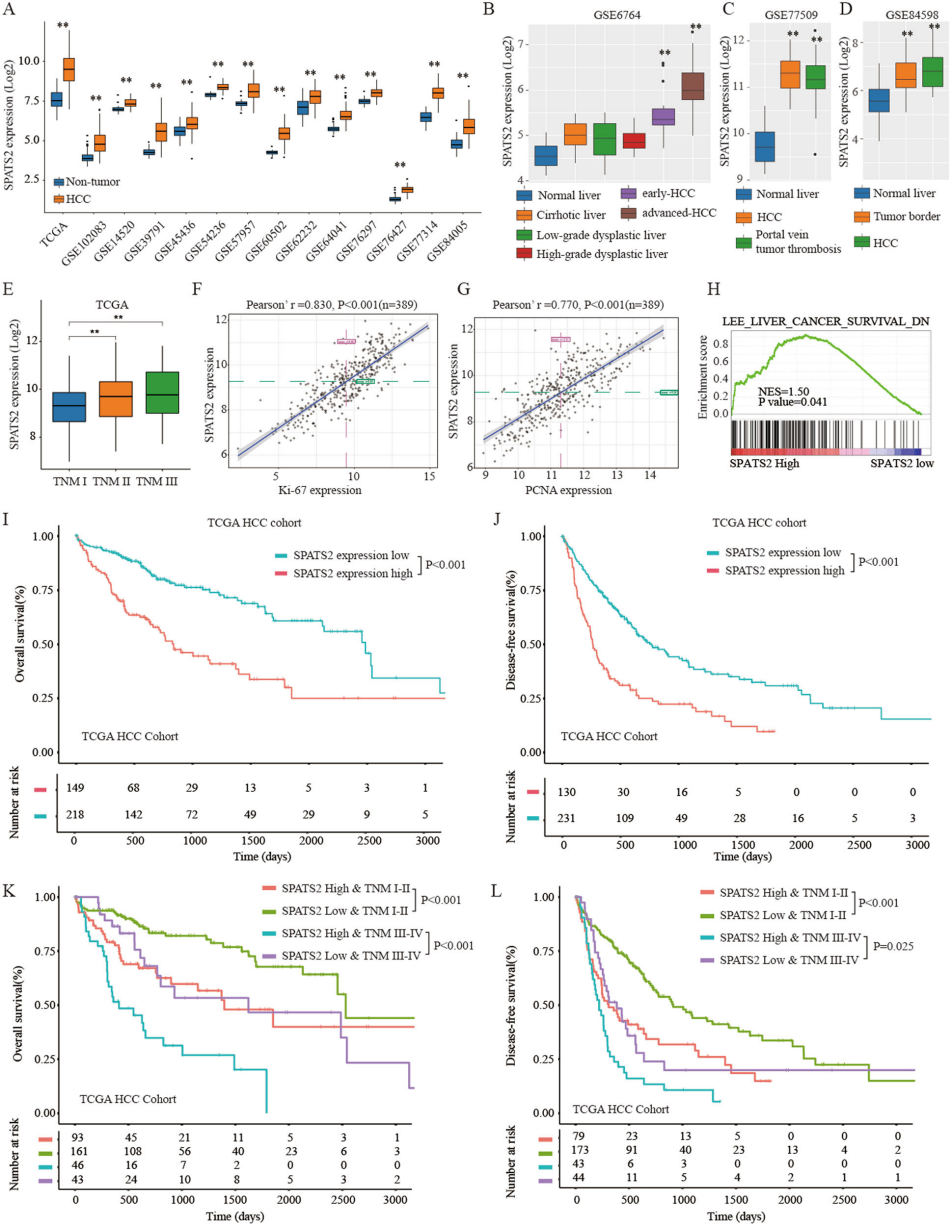
Bioinformatics analysis of the expression and prognostic value of SPATS2 in the TCGA HCC cohort and GEO database. (**a**) Bioinformatics analysis of SPATS2 expression in HCC or non-tumor tissues based on the dataset from TCGA and GEO database. (**b**) Bioinformatics analysis of SPATS2 expression in normal liver, cirrhotic liver, low- or high-grade dysplastic liver, early or advanced HCC in GSE6764 dataset. (**c**) Bioinformatics analysis of SPATS2 expression in normal liver, HCC, and portal vein tumor thrombosis in the GSE77509 dataset. (**d**) Bioinformatics analysis of SPATS2 expression in normal liver, tumor border, and HCC in the GSE84598 dataset. (**e**) Bioinformatics analysis of SPATS2 expression in HCC tissues with different TNM stages based on the dataset from TCGA. (**f**, **g**) Pearson correlation analysis between SPATS2 expression and Ki-67 expression (Pearson’s r = 0.830, *p* < 0.001) or PCNA expression (Pearson’s r = 0.770, *p* < 0.001). (**h**) GSEA analysis of the enrichment of liver cancer survival genes between SPATS2 high expression and low expression groups. (**i, j**) Kaplan–Meier analysis of the overall survival (OS) and disease-free survival (DFS) of HCC patients with high or low SPATS2 expression in TCGA HCC cohort. (**k**, **l**) Kaplan–Meier analysis of OS and DFS of HCC patients with high or low SPATS2 expression at different TNM stages in TCGA HCC cohort. survival. *p < 0.05, **p < 0.01.

We further validated the expression of SPATS2 in TCGA and the GEO database by bioinformatics analysis. As shown in Fig. 2a, compared with the adjacent non-tumor tissues, HCC tissues had remarkably higher expression levels of SPATS2 in all the HCC expression datasets. Moreover, advanced HCC had the highest level of SPATS2 in comparison to that in early HCC, high-grade dysplastic liver, low-grade dysplastic liver, cirrhotic liver, and normal liver (GSE6764, Fig. 2b). Additional results from GSE77509 and GSE84598 analysis showed that HCC/Portal vein tumor thrombosis (Fig. 2c) or HCC/Tumor border (Fig. 2d) had significantly higher expression of SPATS2 that that in normal liver.

In addition, high expression of SPATS2 was positively correlated with advanced HCC TNM stages (Fig. 2e). The Pearson association analysis demonstrated that SPATS2 expression was positively associated with the expression levels of Ki-67 and PCNA in the TCGA dataset (Fig. 2f, g). Gene Set Enrichment Analysis (GSEA) revealed that the liver cancer survival gene set was enriched in the SPATS2 high expression group (Fig. 2h).

Prognostic analysis of TCGA HCC cohort demonstrated that HCC patients with high SPATS2 expression had worse overall survival (OS) and disease-free survival (DFS) than HCC patients with low SPATS2 expression (Fig. 2i, j). Moreover, high SPATS2 expression in HCC patients with different TNM stages (TNM I-II or TNM III-IV) also had markedly lower rates of OS and DFS that in patients with low SPATS2 expression (Fig. 2k, l). In summary, these results indicated high SPATS2 expression was correlated with worse prognosis of HCC patients.

### Knockdown of SPATS2 inhibits the malignant phenotypes of HCC cell lines

**Fig. 3:**
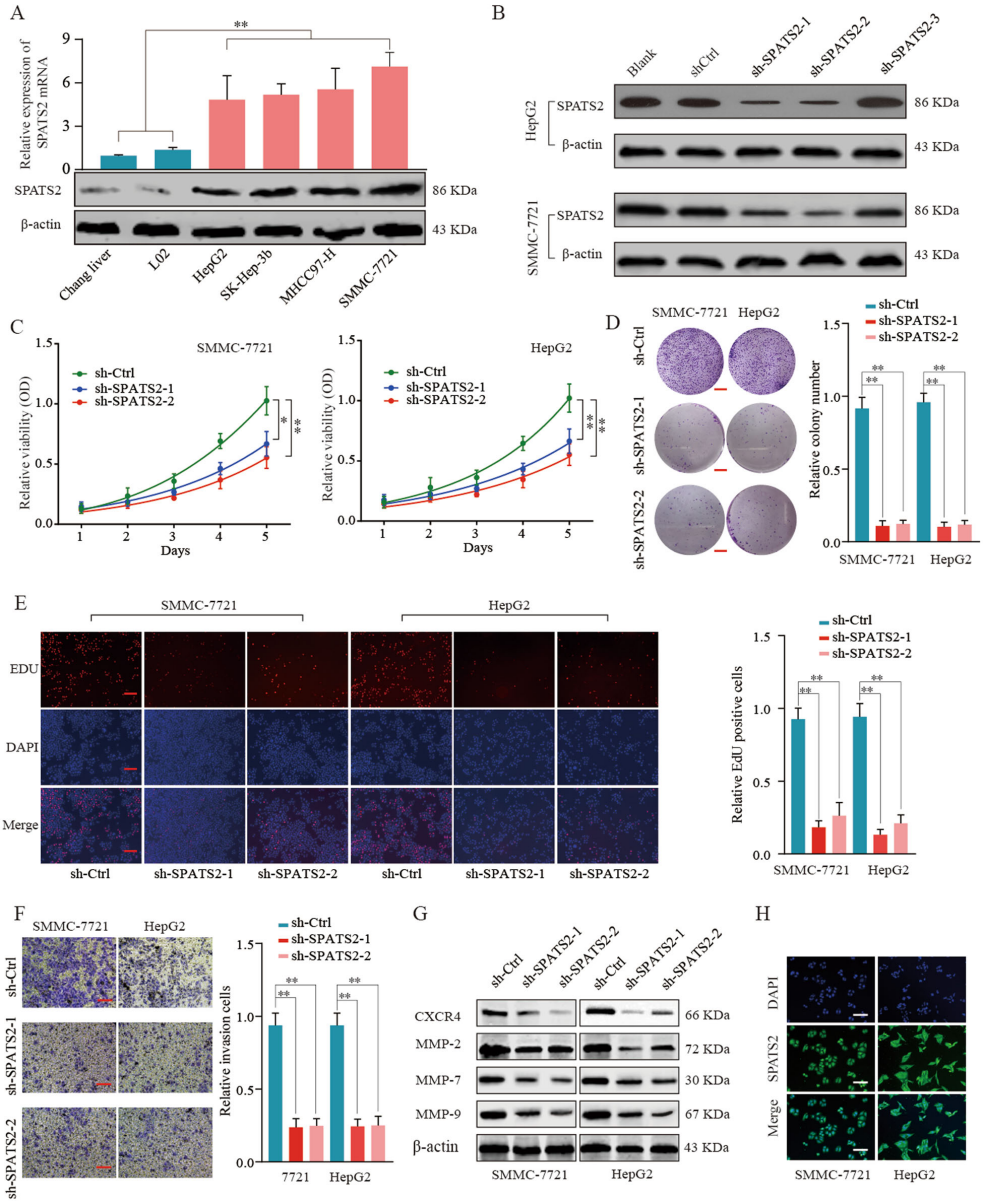
Knockdown of SPATS2 inhibits the malignant phenotypes of HCC cell lines. **a** The mRNA and protein expression levels of SPATS2 in normal liver cell lines (Chang liver and L02) and HCC cell lines (HepG2, SK-Hep-3b, MHCC97-H, and SMMC-7721) were analyzed by RT-qPCR (up panel) and western blot (lower panel). **b** HepG2 or SMMC-7721 cells were left untreated (Blank), transfected with negative control (shCtrl), or transfected with shRNAs targeting SPATS2 (sh-SPATS2-1/2/3). The expression of SPATS2 protein was analyzed by western blot 48 h later. The representative result of three independent experiments was shown. **c** Cell proliferation of SMMC-7721 or HepG2 cells transfected with sh-Ctrl or sh-SPATS2-1/2 was determined by CCK-8 assay at indicated time points. **d** Colony formation assay was performed to determine the colony formation capability of SMMC-7721 or HepG2 cells transfected with sh-Ctrl or sh-SPATS2-1/2. **e** Representative immunofluorescence photos (left) and quantitation of relative EDU positive cells of SMMC-7721 or HepG2 cells transfected with sh-Ctrl or sh-SPATS2-1/2. **f** Transwell experiment was performed to analyze the cell invasion of SMMC-7721 or HepG2 cells transfected with sh-Ctrl or sh-SPATS2-1/2. **g** SMMC-7721 or HepG2 cells were transfected with sh-Ctrl or sh-SPATS2-1/2 and migration-related proteins CXCR4, MMP2, MMP7, and MMP9 expression were analyzed by western blot. **h** SPATS2 and DAPI immunofluorescence staining were performed to verify the localization of SPATS2 protein in HepG2 or SMMC-7721 cells. *p < 0.05, **p < 0.01.

To better understand the role of SPATS2 in HCC, we examined the expression of SPATS2 in different HCC cell lines. The expression of SPATS2 was significantly upregulated in four HCC cell lines (HepG2, SK-Hep-3b, MHCC97-H, and SMMC-7721) in comparison with normal cell lines (Chang liver and L02) (Fig. 3a). HepG2 and SMMC-7721, which had relatively higher SPATS2 expression, were selected for subsequent experiments.

We performed loss-of-function studies using shRNAs targeting SPATS2 and the RNA silence efficiency was determined by western blot (Fig. 3b). Two efficient shRNAs targeting SPATS2 (sh-SPATS2-1 and sh-SPATS2-2) were selected. As shown in Fig. 3c–e, SPATS2 knockdown notably inhibited cell proliferation, colony formation, and DNA synthesis of SMMC-7721 and HepG2 cells. In addition, SPATS2 knockdown significantly decreased the invasion and migration capability of HCC cells (Fig. 3f, Supplementary Fig. 2). Furthermore, knockdown of SPATS2 dramatically inhibited the expression of migration-related proteins, such as CXCR4, MMP-2, MMP-7, and MMP-9, which was consistent with the decreased invasive ability of HCC cells (Fig. 3g). Meanwhile, immunofluorescence-staining results showed that SPATS2 was mostly distributed in cell cytoplasm of HCC cells (Fig. 3h). In contrast, gain-of-function experiments indicated upregulation of SPATS2 promoted HCC cell proliferation and invasion in vitro (Supplementary Fig. 3). Taken together, our loss- and gain-function experiments results demonstrate that SPATS2 exhibits oncogenic function in HCC cells and enhances the malignant phenotypes of HCC.

### Knockdown of SPATS2 inhibits HCC development in vivo

**Fig. 4:**
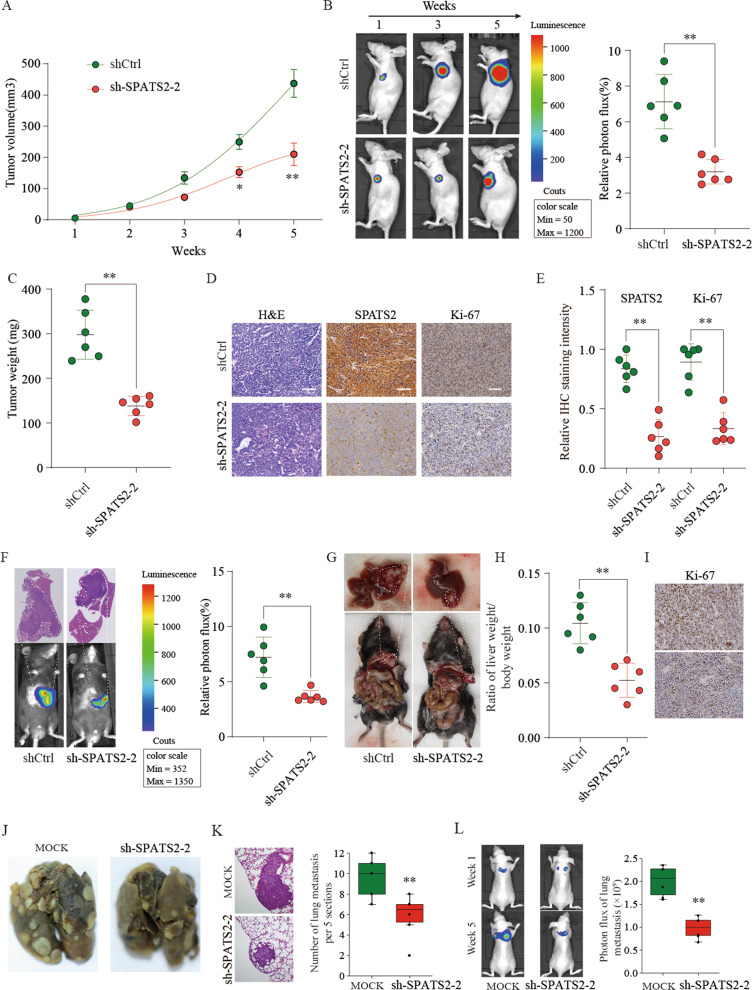
Knockdown of SPATS2 inhibits HCC development in vivo on subcutaneous xenotransplanted, orthotopic implanted, and lung metastasis tumor models. shCtrl or sh-SPATS2-transfected SMMC-7721 cells were implanted subcutaneously into nude mice. (**a**) Tumor volume and growth curves of tumor were monitored every week. **b** The relative photon flux of HCC tumor in nude mice from the shCtrl or sh-SPATS2-2 group was determined by a live imaging system to measure the luciferase signal. **c** The weight of the tumors in nude mice of shCtrl or sh-SPATS2-2 group was measured after sacrifice. **d** Representative H&E staining and IHC staining image of SPATS2 and Ki-67 of tumor sections from shCtrl or sh-SPATS2-2 group. Scale bar = 200 μm. **e** Quantification of relative SPATS2 and Ki-67 staining intensity in tumor sections from the shCtrl or sh-SPATS2-2 group. In HCC orthotopic model, shCtrl or sh-SPATS2-2-transfected Hepa1-6 cells were injected into one liver lobe of C57BL/6 mice. **f** The relative photon flux of HCC orthotopic tumor in nude mice from the shCtrl or sh-SPATS2-2 group was analyzed. **g** The representative HCC orthotopic tumor was shown. **h** The ratio of liver weight/body weight of the shCtrl or sh-SPATS2-2 group was analyzed. **i** Representative IHC staining image of Ki-67 of HCC orthotopic tumor sections from the shCtrl or sh-SPATS2-2 group. Representative H&E stained images of lung metastatic loci from each group and statistical analysis of the number of intrahepatic metastases in each group (**j**, **k**). Images of lung metastases that developed in MHCC97H-sh-SPATS2 lateral tail vein injection models by the IVIS Imaging System. Representative luciferase signals captured in each group at the initial injection and 48 days after the injection of the cells are shown. The statistical analysis is shown (**l**). *p < 0.05, **p < 0.01.

The subcutaneous xenotransplanted and orthotopic implanted tumor models were employed to further investigate the function of SPATS2 on HCC proliferation in vivo. As shown in Fig. 4a–c, the HCC xenograft model showed that SPATS2 knockdown markedly dampened HCC tumor development, with reduced tumor volume and tumor weight. Furthermore, SPATS2 and Ki-67 IHC staining results confirmed that knockdown of SPATS2 drastically decreased the expression of SPATS2 and Ki-67 (Fig. 4d, e). The orthotopic implanted tumor model also confirmed that SPATS2 knockdown markedly decreased the HCC tumor growth with a significantly lower ratio of liver weight/body weight (Supplementary Fig. 4, Fig. 4f–h). We also demonstrated that liver tumor tissues from the sh-SPATS2-2 group had a relatively lower expression of Ki-67 (Fig. 4i).

To further verify the metastasis-promoting role of SPATS2 in vivo, SPATS2-silencing MHCC97H cells (MHCC97H-sh-SPATS2) or negative control (MHCC97H-sh-NC) cells were injected into the lateral tail vein of nude mice. Decreased and smaller micrometastatic lesions were detected microscopically in the lungs of nude mice injected with SPATS2-silenced MHCC97H-sh-SPATS2 cells compared with that in mice injected with MHCC97H-sh-NC cells (Fig. 4j–l). Together, these results suggest that SPATS2 could promote HCC tumor growth and metastasis in vivo.

### SPATS-2 regulates cell apoptosis and cell cycle in HCC

**Fig. 5:**
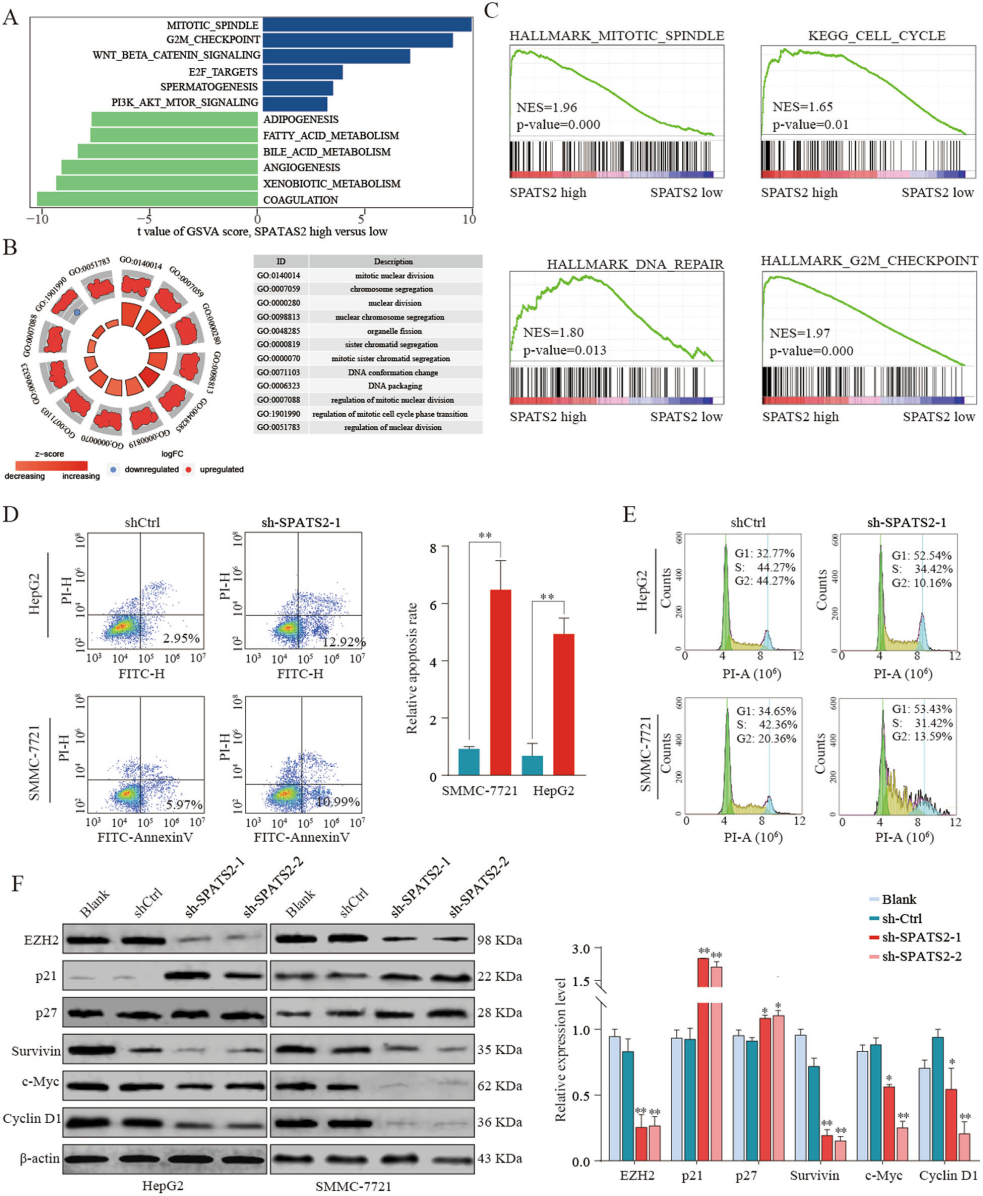
SPATS-2 regulates cell apoptosis and cell cycle in HCC. GSVA (**a**) and GO (**b**) analysis the different regulated signaling pathways in HCC tumor compared with adjacent non-tumor tissues. **c** GSEA analysis the enrichment of signature genes involved in Mitotic spindle, cell cycle, DNA repair and G2M checkpoint in SPATS2 high or low expression group. HepG2 or SMMC-7721 cells were transfected with shCtrl or sh-SPATS2-1. **d** Cell apoptosis and (**e**) cell cycle were analyzed by Annexin V/PI staining. **f** HepG2 or SMMC-7721 cells were left untreated (Blank), or transfected with shCtrl or sh-SPATS2-1/2. The protein expression of cyclin kinase inhibitor p21 and p27, EZH2, survivin, c-myc, and cyclin D1 were analyzed by western blot. The experiment was repeated three times and the representative blot image was shown. *p < 0.05, **p < 0.01.

To further explore the biological function of SPATS2 in HCC, comprehensive bioinformatics analysis was performed using TCGA HCC datasets. The results showed that cell cycle related pathways were significant activated in SPATS2-high HCC tissues compared with those in non-malignant tissues (Supplementary Fig. 5A–C, Fig. 5a, b). Moreover, GSEA analysis showed that mitotic spindle (NES = 1.96, *p* = 0.000), cell cycle (NES = 1.65, *p* = 0.01), DNA repair (NES = 1.8, *P* = 0.013), and G2M checkpoint (NES = 1.97, *P* = 0.000) were significantly enriched in SPATS2 high group (Fig. 5c). Considering the potential role of SPATS2 in DNA repair and cell cycle, the function of SPATS2 on cell apoptosis and cell cycle was explored. Intriguingly, SPATS2 knockdown significantly induced cell apoptosis and G1 arrest of HepG2 or SMMC-7721 cells (Fig. 5d, e). Moreover, protein expression of cyclin kinase inhibitor p21 and p27 was significantly upregulated in SPATS2-silencing SMMC-7721 cells, while only upregulated p21 was observed in SPATS2-silencing HepG2 cells (Fig. 5f). In addition, EZH2, survivin, c-myc, and cyclin D1 were markedly downregulated in the SPATS2 knockdown group (Fig. 5f). The apoptosis promoted effect induced by SPATS2 silencing was further confirmed by Tunel assay (Supplementary Fig. 6A). Moreover, anti-apoptotic protein Bcl-2 was significantly downregulated in the SPATS2 knockdown group compared with that in control group, while pro-apoptotic protein Bax, Bak, cleaved-caspase 3, and cytochrome C were remarkably upregulated after SPATS2 knockdown (Supplementary Fig. 6B). In summary, SPATS2 plays an important role in cell apoptosis and cell cycle processes in HCC.

### MiR-145-5p negatively regulates SPATS2 expression by binding to the 3′-UTR of SPATS2

**Fig. 6:**
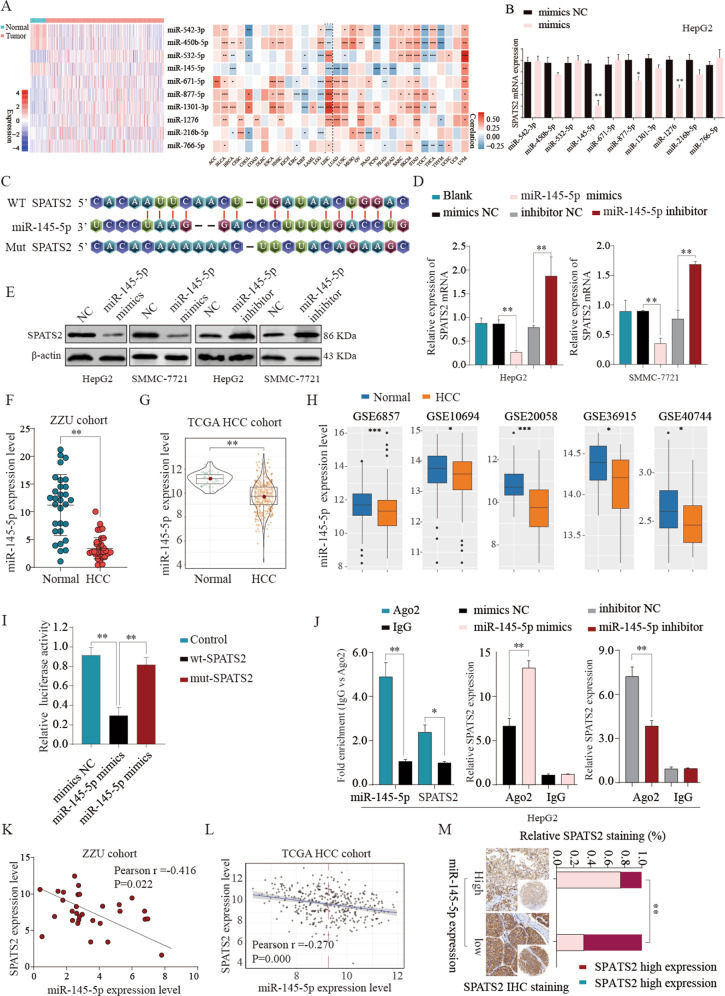
MiR-145-5p inhibits SPATS2 expression by targeting 3’-UTR of SPATS2. **a** The expression status of candidate miRNAs targeting SPATS2 was analyzed based on the TCGA database (left panel). Pearson analysis of the correlation between SPATS2 and candidate miRNAs based on the TCGA database (right panel). **b** Expression of SPATS2 was detected under the treatment of candidate miRNA mimics in Hep-G2 cells. **c** Diagram of the putative binding of MiR-145-5p on the 3′-UTR of SPATS2 and the mutated sequences of SPATS2 3′-UTR. The expression levels of SPATS2 mRNA or protein in HepG-2 or SMMC-7721 cells transfected with MiR-145-5p mimics, MiR-145-5p inhibitor, or relative negative controls were analyzed by RT-qPCR (**d**) or western blot (**e**), respectively. The expression levels of MiR-145-5p in HCC tissues and normal control tissues were analyzed in ZZU (**f**), TCGA (**g**), and GEO (**h**) HCC cohorts. The representative result of at least three independent experiments was shown. **i** WT 3′-UTR or mutated 3′-UTR of SPATS2 was constructed into luciferase reporter vector and co-transfected with MiR-145-5p mimics into HEK293 cells. Relative luciferase activity was determined 48 h after transfection. **j** RIP assays confirmed the binding status between MiR-145-5p and SPATS2 in untreated and treated HCC cell lines, respectively. **k**, **l** Pearson correlation analysis of the MiR-145-5p expression and SPATS2 expression in ZZU and TCGA HCC cohort. **m** Representative IHC staining of SPATS2 in MiR-145-5p high- or low- HCC tissues from ZZU cohort. *p < 0.05, **p < 0.01.

To investigate how SPATS2 expression was regulated in HCC, 10 candidate miRNAs targeting SPATS2 were selected based on the online bioinformatic database (StarBase 3.0) and expression status (Fig. 6a). Further validation experiments revealed that miR-145-5p overexpression significantly decreased the SPATS2 mRNA levels (Fig. 6b). As shown in Fig. 6c, MiR-145-5p was predicted to have the complementary binding sequences mapping to the 3′-UTR of SPATS2. While MiR-145-5p mimics could significantly decrease the mRNA and protein expression levels of SPATS2, MiR-145-5p inhibitor significantly enhanced SPATS2 expression in HepG2 or SMMC-7721 cells (Fig. 6d, e). The expression of MiR-145-5p was notably downregulated in HCC tissues in comparison with that in normal tissues in ZZU, TCGA, and GEO cohorts (Fig. 6f–h; Supplementary Fig. 7). Additionally, MiR-145-5p mimics specifically inhibited the luciferase activity of luciferase reporter vector with WT 3′-UTR of SPATS2, not mutated 3′-UTR of SPATS2 (Fig. 6i). We further performed RIP to explore the binding status between MiR-145-5p and SPATS2 in HCC cell lines. RIP assay showed that MiR-145-5p and SPATS2 were both enriched in Ago2-coating beads relative to the IgG control group. SPATS2 is significant upregulated in HCC cells transfected with MiR-145-5p-mimics, while reverse changes of SPATS2 were observed in HCC cells transfected with MiR-145-5p-inhibitor (Fig. 6j). Furthermore, MiR-145-5p was negatively correlated with SPATS2 expression in both ZZU and TCGA HCC cohort (Fig. 6k, l). In addition, MiR-145-5p was inversely associated with the IHC staining intensive of SPATS2 in HCC tissues (Fig. 6m). In summary, these data suggest that MiR-145-5p might directly inhibit the expression of SPATS2.

### MiR-145-5p inhibits HCC cell proliferation and invasion in vitro by controlling SPATS2

**Fig. 7:**
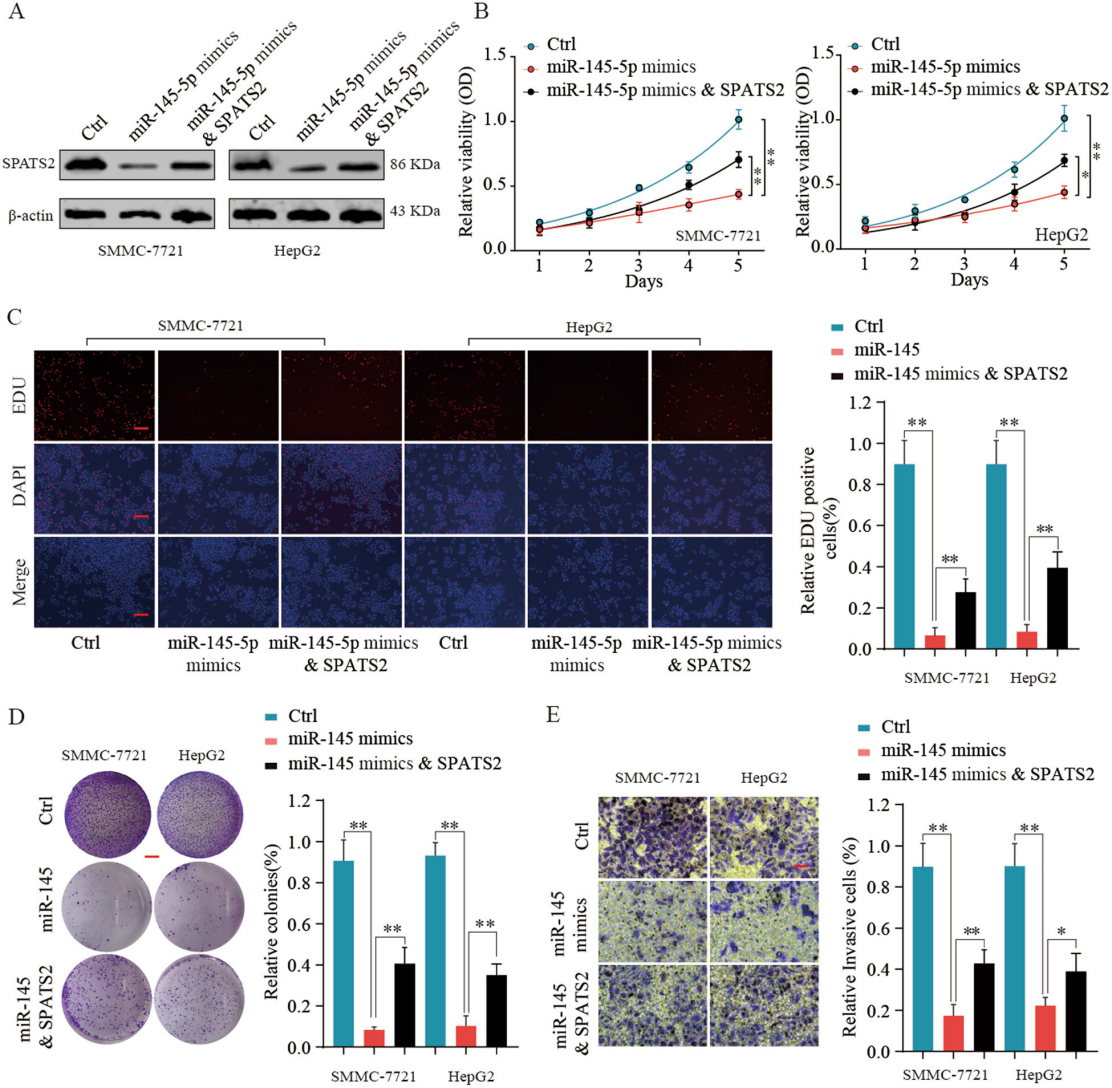
MiR-145-5p inhibits HCC cell proliferation and invasion in vitro by controlling SPATS2. HCC cells SMMC-7721 or HepG2 were transfected with negative control (Ctrl), MiR-145-5p mimics, or MiR-145-5p mimics & SPATS2. **a** The protein expression levels of SPATS2 in different groups were analyzed by western blot 48 h later. The representative result of at least three independent experiments was shown. **b** Cell viability was assessed at indicated time points by CCK-8 assay. **c** EDU incorporation, (**d**) colony formation, and (**e**) transwell assays were performed to evaluate the HCC cell proliferation and invasion in vitro. Results were shown as mean ± SD. *p < 0.05, **p < 0.01.

**Fig. 8:**
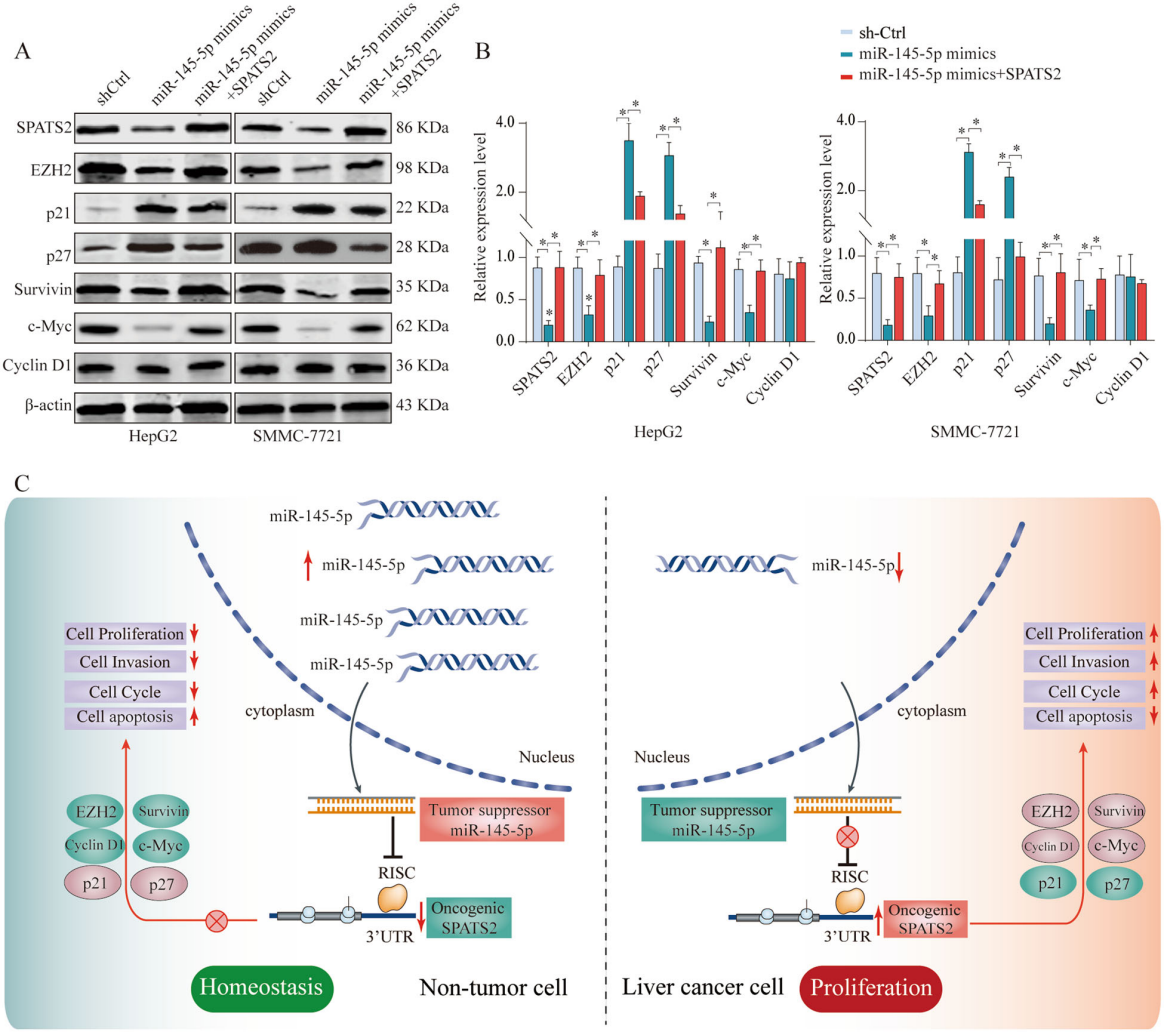
MiR-145-5p/SPATS2 axis regulates cell apoptosis and cell cycle in HCC. HCC cells SMMC-7721 or HepG2 were transfected with negative control (Ctrl), MiR-145-5p mimics, or MiR-145-5p mimics & SPATS2. **a**, **b** The protein expression of SPATS2, EZH2, p21, p27, survivin, c-myc, and cyclin D1 were analyzed by western blot. The experiment was repeated three times and the representative blot image was shown. **c** Illustrated diagram of the MiR-145-5p/SPATS2 axis working model in HCC. Results were shown as mean ± SD. *p < 0.05.

To further determine the functional relationship between SPATS2 and MiR-145-5p in regulating HCC progression, rescue experiments were executed. Overexpression of MiR-145-5p significantly inhibited SPATS2 expression in SMMC-7721 or HepG2 cells. However, co-transfection MiR-145-5p with SPATS2 overexpression vector restored SPATS2 expression (Fig. 7a). Functionally, while MiR-145-5p mimics significantly suppressed cell proliferation and invasion of SMMC-7721 or HepG2 cells, overexpression SPATS2 antagonized the inhibition effects of MiR-145-5p mimics (Fig. 7b–e, Supplementary Fig. 8). Cell cycle related p21 and p27 expression were significantly upregulated in MiR-145-5p mimic transfected HepG2 or SMMC-7721 cells, but the upregulation was partly reversed in the MiR-145-5p&SPATS2 group (Fig. 8a, b). In addition, the inhibition of SPATS2, EZH2, Survivin, and c-Myc expression by MiR-145-5p was partially reversed by SPATS2 overexpression (Fig. 8a, b).

Moreover, low expression of miR-145-5p in HCC tissues significantly predicted unfavorable prognosis in the TCGA dataset (Supplementary Fig. 9A, B**)**, and this tendency was more remarkable in patients with miR-145-5p^low^/PGAM1^high^ profiles (Supplementary Fig. 9C and 9D). Overall, our results suggest that miR145 might exert its function via regulating SPATS2 expression. (Fig. 8c).

## Discussion

Although accumulating studies have revealed the potential oncogenic function of SPATS2 in colorectal cancer and squamous cell carcinoma, the role of SPATS2 in HCC has not been investigated so far^6,7^. In this study, we found that SPATS2 was upregulated in HCC tissues in comparison with that in adjacent normal tissues. Furthermore, high SPATS2 expression was positively correlated with vascular invasion, TNM stage, tumor size, and poor OS/DFS rate. The results indicated that SPATS2 might be an oncogene in HCC. Moreover, we found that SPATS2 markedly promoted HCC cell proliferation and invasion through downregulating the expression of p21 and p27, while upregulating the expression of EZH2, survivin, c-myc, and cyclin D1. SPATS2 knockdown significantly inhibited tumor progression in vivo. The results indicate SPATS2 may function as a tumor-promoting oncogene in HCC.

To further explore the molecular mechanism of SPATS2 in HCC development, we revealed that SPATS2 participated in the proliferation of HCC cells by regulating cell cycle by bioinformatics analysis. P21 and p27 were well-known tumor suppressors, which could induce cell cycle arrest^25,26^. A previous study demonstrated that EZH2 was an oncogene promoting HCC cell proliferation via Wnt/β-catenin signaling pathway^27^. Wang et al. demonstrated that survivin could regulate cell cycle and suppress HCC cell apoptosis^28^. Similarly, oncogenic c-Myc suppressed sorafenib-induced HCC cell apoptosis^29^. In addition, cyclin D1 was reported to promote the development of HCC through regulating cell cycle^30^. Here, we found that the expression of p21 and p27 were upregulated in the SPATS2 knockdown group compared with that in control group. Meanwhile, EZH2, survivin, and c-myc were downregulated after SPATS2 knockdown. However, we did not notice any significant changes of cyclin D1 after SPATS2 knockdown. Nevertheless, SPATS2 might regulate the cell cycle through modulating the expression of other critical cell cycle regulators, such as CDK2, CDK4, CDK6, or cyclin E, which needs further investigation.

Accumulating evidence indicates that miRNAs are crucial regulators of tumor proliferation and invasion. For instance, miR-1258 inhibited proliferation, migration, and invasion of non-small-cell lung cancer cell lines by downregulation of GRB2^31^. MiR-9 inhibited gastric cell proliferation, invasion by downregulating TNFAIP8^32^. In this study, using bioinformatics prediction tools, we identified that MiR-145-5p could directly interact with SPATS2 by binding to its 3′-UTR and there was a negative association between MiR-145-5p and SPATS2 expression in HCC. MiR-145-5p overexpression could significantly decrease SPATS2 expression while MiR-145-5p inhibitor could enhance SPATS2 expression both at mRNA and protein levels. Functionally, MiR-145-5p overexpression remarkably inhibited the proliferation and invasion of HCC cells. However, overexpression SPATS2 together with MiR-145-5p partially reversed the inhibitory effect of MiR-145-5p overexpression. These results demonstrate that MiR-145-5p might inhibit the proliferation and invasion of HCC by regulating SPATS2.

In conclusion, our results find that SPATS2 is upregulated in HCC tissues and cell lines. Knockdown of SPATS2 dampens HCC development and metastasis by regulating cell cycle and cell apoptosis. Furthermore, SPATS2 is a direct target of MiR-145-5p and MiR-145-5p exerts its tumor-suppressive function via negatively regulating SPATS2 expression. Taken together, our results provide a profound insight of the SPATS2/MiR-145-5p axis in understanding HCC development and progression, which might be used as a promising therapeutic target of HCC.

## Materials and methods

### Human specimens

Fresh HCC samples and corresponding peritumor tissues were collected from patients diagnosed with HCC between May 2014 and March 2016 at the First Affiliated Hospital of Zhengzhou University (Henan, China). Tissue microarrays (TMA) with 396 paired HCC tissues (341 with available follow-up data) and matched non-tumor control tissues were obtained from HCC patients undergoing surgery as described previously^12^. The study was approved by the First Affiliated Hospital of Zhengzhou University Institutional Review Board and all patients signed informed consent.

### Dataset analysis

Public dataset acquisition and process were conducted as previously described^33^. The Cancer Genome Atlas (TCGA) database (http://gdc-portal.nci.nih.gov/) and Genotype-Tissue Expression (GTEX) database (https://gtexportal.org/) were used to analyze the expression of SPATS2 in pan-cancers. Sixteen sets of mRNA microarrays and seventeen miRNA microarrays were downloaded from the Gene Expression Omnibus (GEO) database (http://www.ncbi.nlm.nih.gov/geo/) to analyze the expression of SPATS2 and MiR-145-5p-5p in HCC tissues and normal tissues (Supplementary Table 2,3).

### Cell culture, vector construction, immunofluorescence staining, and Real-time quantitative polymerase chain reaction (RT-qPCR)

These methods were described in the Supplementary Materials and Methods section.

### Immunohistochemistry (IHC)

IHC was conducted as previously described^34^. IHC staining was evaluated by three senior pathologists using Image-Pro Plus software (NIH, USA). The antibodies used in the IHC staining were listed below: SPATS2 (sc-390306, Santa Cruz), ki-67 (27309-1-AP, Proteintech). Sections were semi-quantitatively scored for the SPATS2 IHC staining patterns as follows: the staining extent in each core was scored as 1 + (< 25% staining of cells), 2 + (25–50% staining of tumor cells), 3 + (50 to 75% staining of tumor cells), or 4 + (> 75% staining of tumor cells). Additionally, the staining intensity was quantified as 0 (negative), 1 + (weak), 2 + (intermediate), or 3 + (strong). The final score was obtained by multiplying the intensity and extension values (range 0–12) and the samples were grouped as 1 + (score 0-1), 2 + (score 2-3), 3 + (score 3-4), 4 + (score 6–8) and 5 + (score 9-12) staining. Meanwhile, for statistical purposes, scores of 4+ and 5+ were defined as high expression and the other final scores were considered as low expression.

### Western blotting

Western blot was conducted as previously described^16^. Protein was visualized by ECL plus Detection Reagents (RPN2132; GE Healthcare, UK). The primary antibodies used were listed in Supplementary Table 1.

### CCK-8, EdU, cell invasion, and colony formation assay

These assays were described in the Supplementary Materials and Methods section.

### Cell apoptosis and cell cycle assay

Apoptosis and cell cycle of HepG2 or SMMC-7721 cells from different groups (transfected with different plasmids or miRNAs: sh-SPATS2/control, MiR-145-5p/MiR-145-5p&SPATS2/control) were analyzed with a flow cytometer (Beckman, USA). FITC-Annexin V apoptosis kit (Sungenebiotech, China) was used to evaluate apoptotic HCC cells. For cell cycle analysis, cells from different groups were fixed with 70% pre-chilled ethanol overnight. Then, cells were washed with PBS and stained with 20 μL Propidium iodide (Sigma, USA) before analysis by flow cytometer.

### Luciferase reporter assay and RNA immunoprecipitation assay

These methods are described in the Supplementary Materials and Methods section.

### Subcutaneous and orthotopic HCC xenograft models

The animal experiments were approved by the Institute Ethics Committee of The First Affiliated Hospital of Zhengzhou University. The function of SPATS2 was evaluated by subcutaneous and orthotopic xenograft models in vivo. For the subcutaneous model, sh-SPATS2 or control shRNA-transfected SMMC-7721 cells (3 × 10^6^ in 0.2 ml PBS) were inoculated subcutaneously into the left and right side of the nude mice (male, 18–22 g, *n* = 6 per group). Tumor volume was recorded every day (volume = 0.5 × length × width^2^), and tumor weight was measured after sacrifice. For the orthotopic model, sh-SPATS2 or control shRNA-transfected Hepa1-6 cells (1 × 10^6^ in 0.1 ml PBS) were inoculated into one liver lobe of C57BL/6 (male, 18–22 g, *n* = 6 per group). Body weight was measured after sacrifice. For both models, fluorescent pictures were taken with a vivo imaging system and photon flux was also measured. Tumor tissues were collected for further IHC analysis.

### Statistical analysis

The results were presented as means ± standard deviation. The Student’s *t* test and one-way ANOVA were performed to evaluate the statistical significance. A value of *p* < 0.05 was considered to be statistically significant. Statistical analyses were performed using the SPSS software (V16.0, SPSS, Chicago, USA).

## Supplementary information

Supplementary Figure Legends

Supplemently Figure 1

Supplemently Figure 2

Supplemently Figure 3

Supplemently Figure 4

Supplemently Figure 5

Supplemently Figure 6

Supplemently Figure 7

Supplemently Figure 8

Supplemently Figure 9

supplementary table 1-3

Supplementary materials and methods
