# Supplementary material for: SPATS2, negatively regulated by miR-145-5p, promotes hepatocellular carcinoma progression through regulating cell cycle
Source: Cell Death Dis. 2020 Oct 9;11(10):837. doi: 10.1038/s41419-020-03039-y (PMC7547105; doi:10.1038/s41419-020-03039-y)
Supplement: Supplementary file 1 — Supplementary Figure Legends [file 41419_2020_3039_MOESM1_ESM.docx]

Supplementary Figures

**Supplementary Figure 1. SPATS2 expression is frequently dysregulated in various cancers.** Bioinformatics analysis of SPATS2 mRNA expression level in TCGA and GTEX pancancer database. SPATS2 was up-regulated in tumor tissues of Adrenocortical carcinoma (ACC), Bladder urothelial carcinoma (BLCA), Breast cancer (BRCA), Cholangiocarcinoma (CHOL), Colon cancer (COAD), DLBC (Lymphoid Neoplasm Diffuse Large B-cell Lymphoma), Esophagus cancer (ESCA), Glioblastoma multiforme (GBM), Head and Neck squamous cell carcinoma (HNSC), Kidney renal clear cell carcinoma (KIRC), Kidney renal papillary cell carcinoma (KIRP), Brain Lower Grade Glioma (LGG), Liver cancer (LIHC), Lung cancer (LUAD), Lung squamous cell carcinoma (LUSC), Ovarian serous cystadenocarcinoma (OV), Pancreatic adenocarcinoma (PAAD), Pheochromocytoma and Paraganglioma (PCPG), Prostate adenocarcinoma (PRAD), Rectal cancer (READ), Sarcoma (SARC), Skin Cutaneous Melanoma (SKCM), Stomach cancer (STAD), Testicular Germ Cell Tumors (TGCT), Thyroid cancer (THCA), Thymoma (THYM) and Uterine Carcinosarcoma (UCS), while low down-regulated in Acute Myeloid Leukemia (LAML). Ns, no significant differences, ***p* < 0.01, ****p* < 0.01.

**Supplementary Figure 2.** **Knockdown of SPATS2 inhibited cell migration as indicated by wound healing assays.** Wound healing assays were performed to determine the migration of SMMC-7721 and HepG2 cells. The experiment was repeated three times and the representative blot image was shown. ***p* < 0.01.

**Supplementary Figure 3.** **In vitro effects of SPATS2 overexpression on cell proliferation and invasion in HCC cells.** (A) HepG2 or SMMC-7721 cells were transfected with negative control plasmid (vector), or transfected with SPATS2 overexpression plasmid (SPATS2). The expression of SPATS2 protein was analyzed by western blot 48 h later. The representative result of three independent experiments was shown. (B) Cell proliferation of SMMC-7721 or HepG2 cells was determined by CCK-8 assay at indicated time points. (C) Colony formation assay was performed to determine the colony formation capability of SMMC-7721 or HepG2 cells transfected with vector or SPATS2 plasmids. (D) Representative immunofluorescence photos (left) and quantitation of relative EDU positive cells of SMMC-7721 or HepG2 cells transfected with vector or SPATS2 plasmids. (E) Transwell experiment was performed to analyze the cell invasion of SMMC-7721 or HepG2 cells transfected. (F) Migration related proteins CXCR4, MMP2, MMP7 and MMP9 expression were analyzed by western blot. Scale bar = 100 μm. The experiment was repeated three times and the representative blot image was shown. **p* < 0.05, ***p* < 0.01.

**Supplementary Figure 4. Transfection efficiency were confirmed in Hepa1-6 cells**

Hepa1-6 cells were untreated (Blank), transfected with negative control (sh-Ctrl), or transfected with shRNAs targeting SPATS2 (sh-SPATS2-1). The expression of SPATS2 protein was analyzed by western blot 48 h later. The representative result of three independent experiments was shown.

**Supplementary Figure 5. The potential molecular mechanism of SPATS2’s function in HCC.**

(A) GO enrichment, (B) KEGG enrichment analysis and Hallmark of cancer pathway analysis of the top 2000 genes with highest SPATS2 correlation coefficient in TCGA HCC dataset.

**Supplementary Figure 6.** **SPATS2 regulates the protein expression of cell apoptosis-related genes in HCC.** HCC cells HepG2 or SMMC-7721 was transfected with shCtrl, sh-SPATS2-1 or sh-SPATS2-2. (A) Representative micrographs and quantification of Tunel positive signaling in the indicated assay. (B) The protein expression level of Bcl-2, Bax, Bak, cleaved-caspase 3, cytochrome C was analyzed 48 h later. The experiment was repeated three times and the representative blot image was shown. Scale bar = 100 μm. **p* < 0.05

**Supplementary Figure 7. MiR-145-5p inhibits HCC cell migration in vitro by controlling SPATS2.** HCC cells SMMC-7721 or HepG2 were transfected with negative control (Ctrl), MiR-145-5p mimics, or MiR-145-5p mimics & SPATS2. Wound healing assays were performed to evaluate the HCC cell migration in vitro. Results were shown as mean ± SD. *p < 0.05, **p < 0.01.

**Supplementary Figure 8. Low miR-145-5p were associated with cancer progression of HCC patients.** Bioinformatics analysis of miR-145-5p expression in HCC tissues with different clinical phenotype based on the dataset from GSE67138, GSE30297 and GSE10694.

**Supplementary Figure 9. miR-145-5p-SPATS2 correlates with HCC patient outcomes**. Kaplan-Meier analysis of the overall survival or disease-free survival in HCC patients according to miR-145-5p expression level (A, B) and concurrent expression of miR-145-5p and SPATS2.
