# Supplementary figures and images for: SPATS2, negatively regulated by miR-145-5p, promotes hepatocellular carcinoma progression through regulating cell cycle
Source: Cell Death Dis. 2020 Oct 9;11(10):837. doi: 10.1038/s41419-020-03039-y (PMC7547105; doi:10.1038/s41419-020-03039-y)

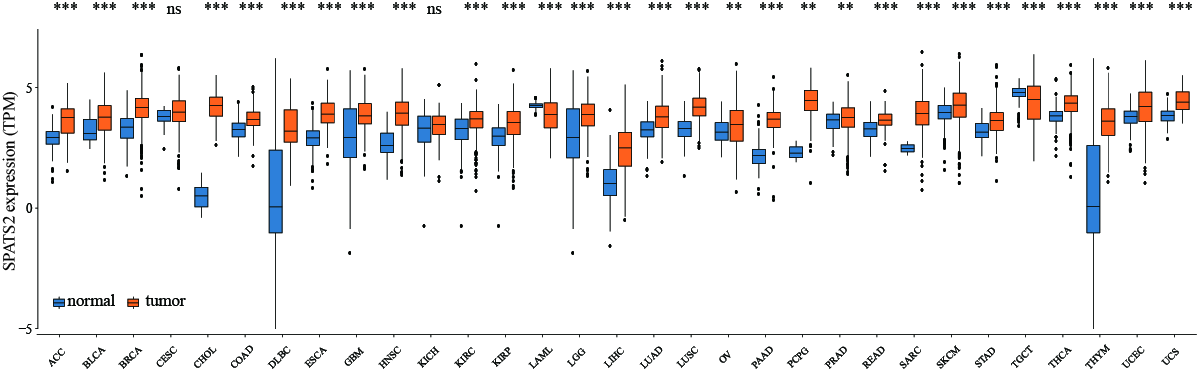

Supplement: Supplementary file 2 — Supplemently Figure 1 [file 41419_2020_3039_MOESM2_ESM.tif]

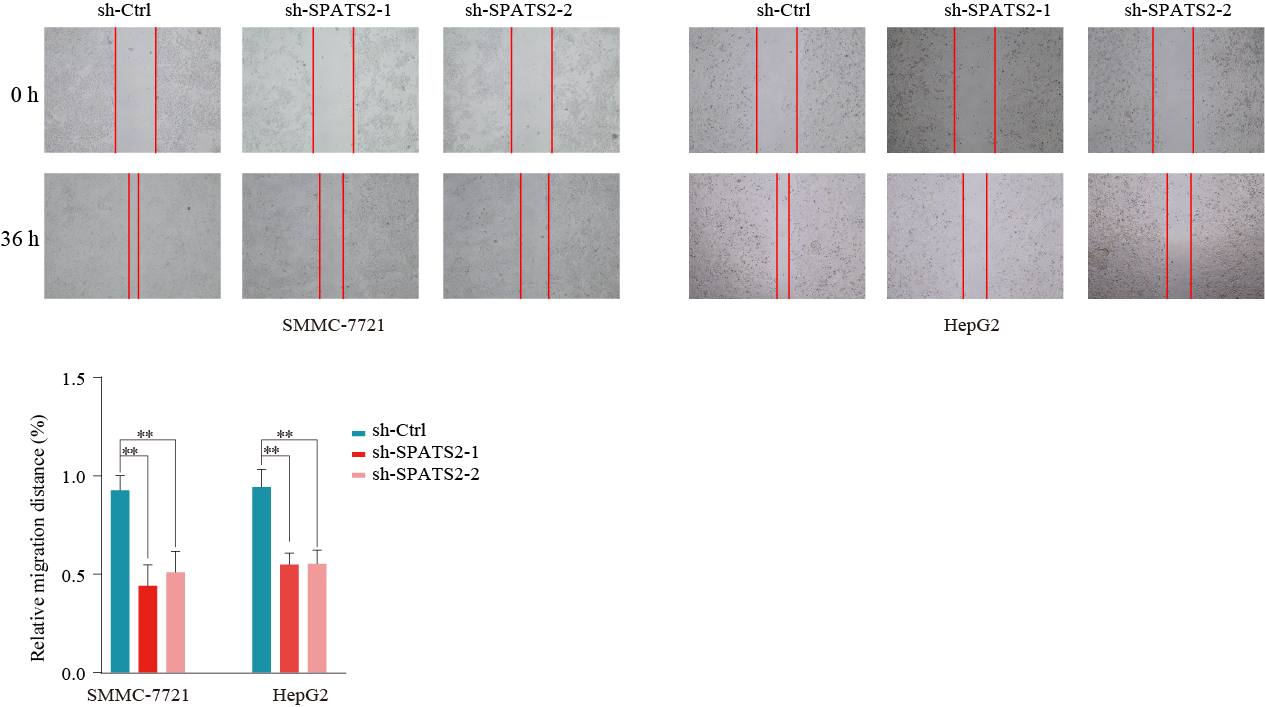

Supplement: Supplementary file 3 — Supplemently Figure 2 [file 41419_2020_3039_MOESM3_ESM.tif]

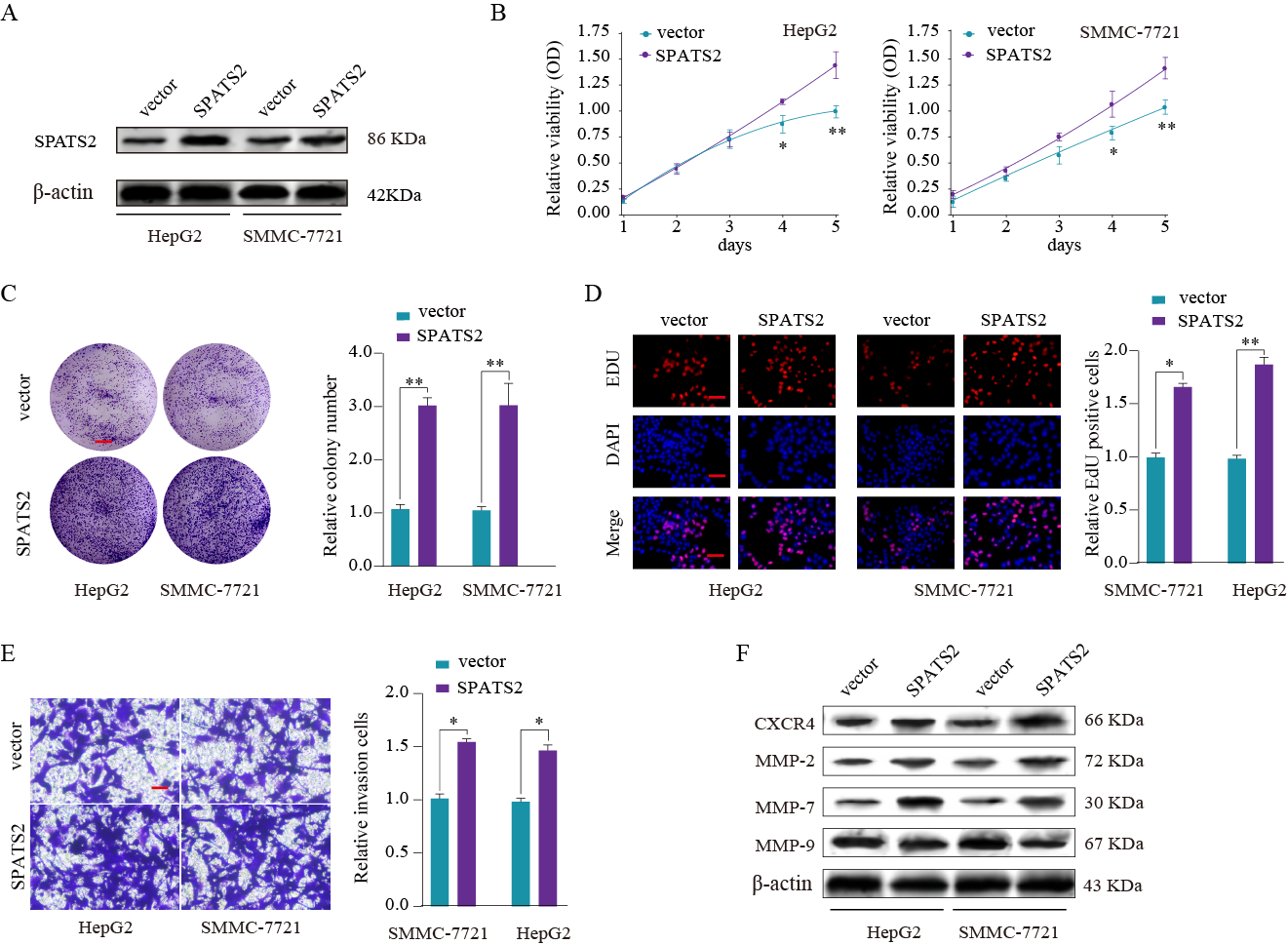

Supplement: Supplementary file 4 — Supplemently Figure 3 [file 41419_2020_3039_MOESM4_ESM.tif]

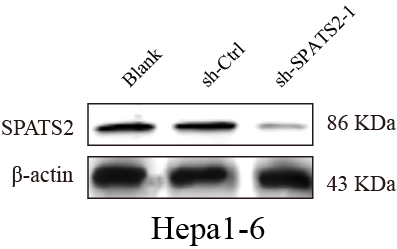

Supplement: Supplementary file 5 — Supplemently Figure 4 [file 41419_2020_3039_MOESM5_ESM.tif]

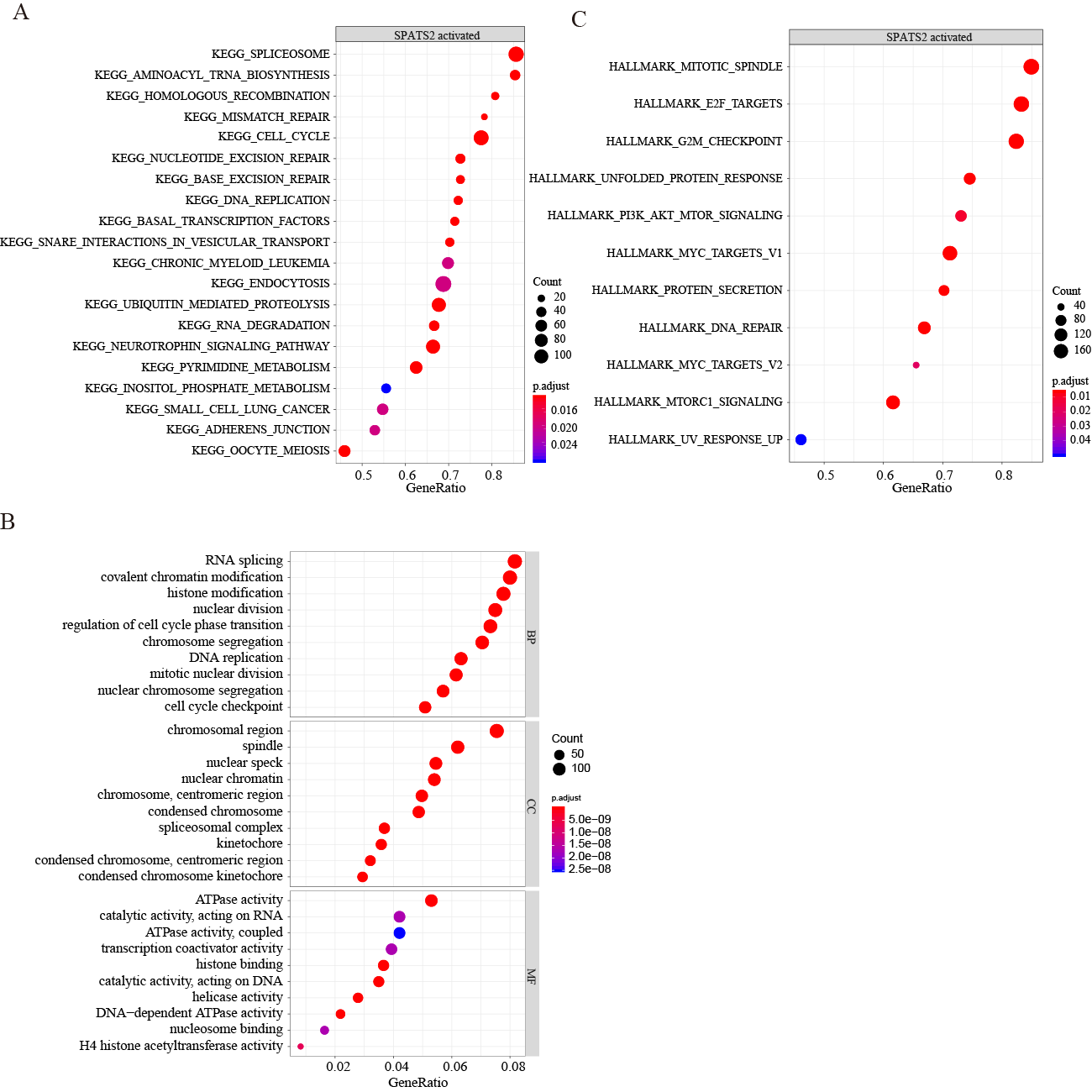

Supplement: Supplementary file 6 — Supplemently Figure 5 [file 41419_2020_3039_MOESM6_ESM.tif]

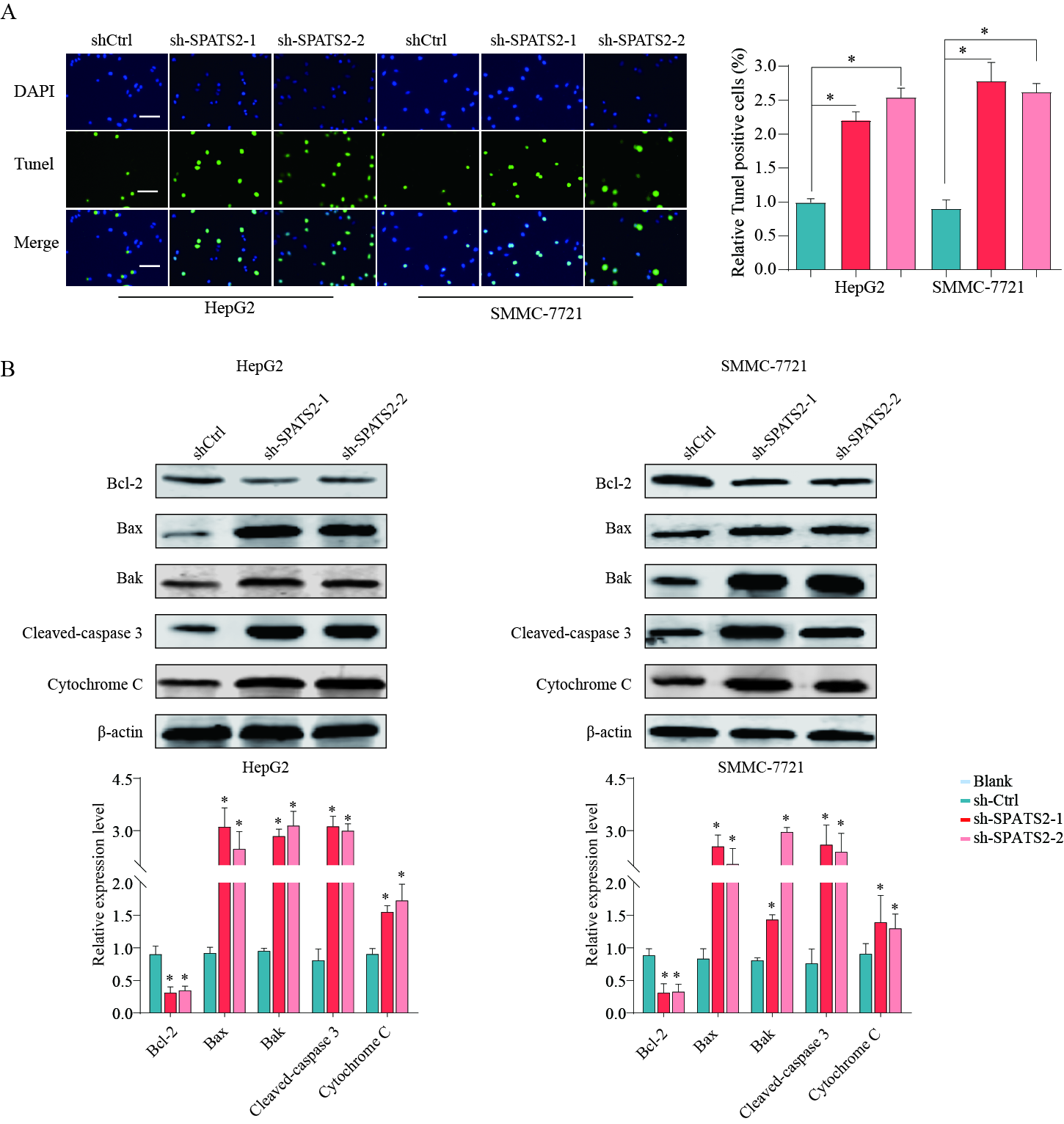

Supplement: Supplementary file 7 — Supplemently Figure 6 [file 41419_2020_3039_MOESM7_ESM.tif]

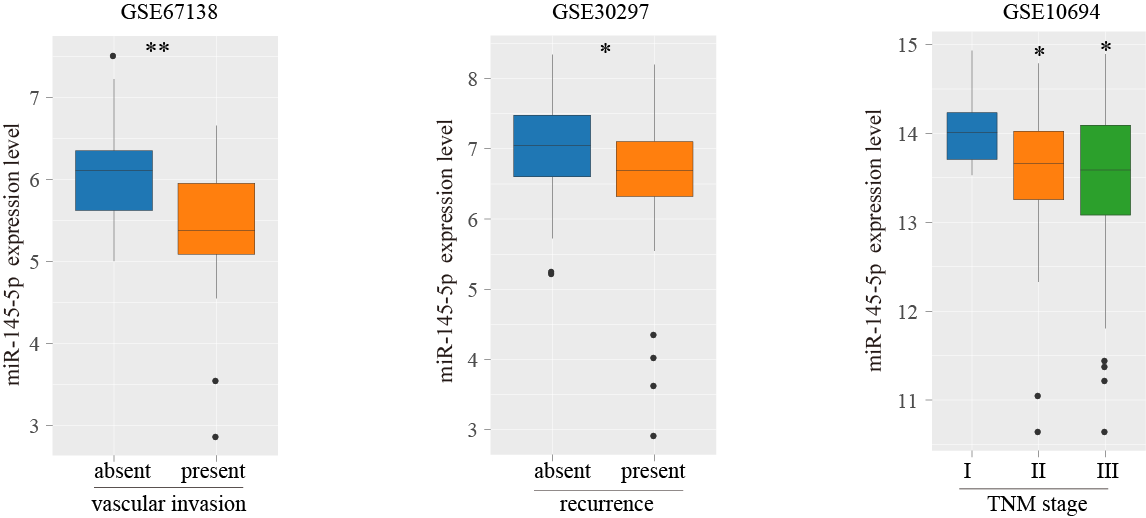

Supplement: Supplementary file 8 — Supplemently Figure 7 [file 41419_2020_3039_MOESM8_ESM.tif]

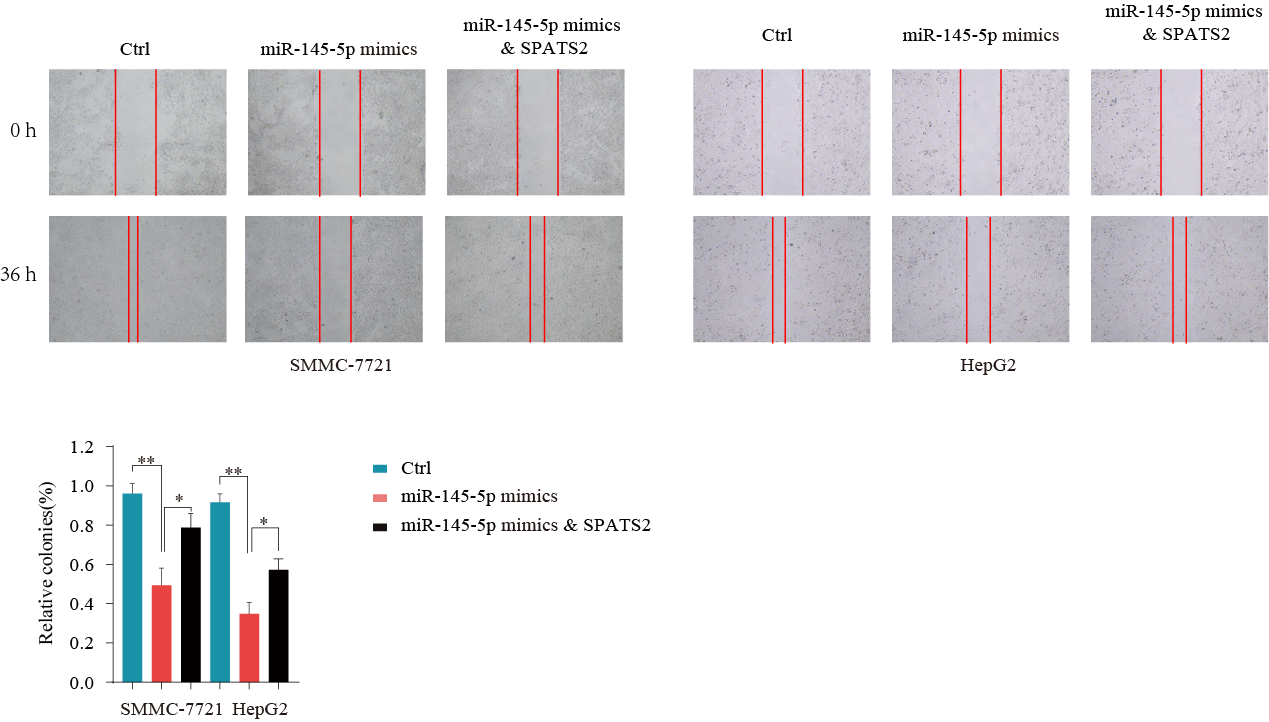

Supplement: Supplementary file 9 — Supplemently Figure 8 [file 41419_2020_3039_MOESM9_ESM.tif]

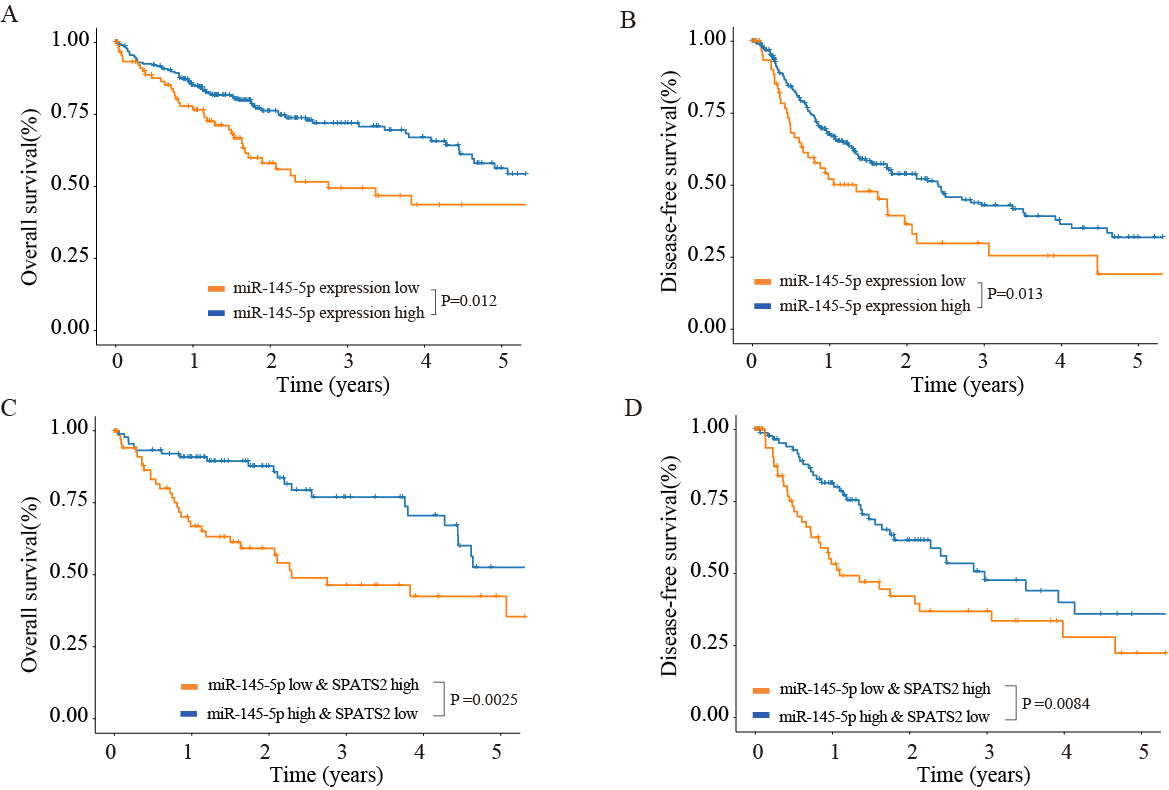

Supplement: Supplementary file 10 — Supplemently Figure 9 [file 41419_2020_3039_MOESM10_ESM.tif]
