# Supplementary material for: SPATS2, negatively regulated by miR-145-5p, promotes hepatocellular carcinoma progression through regulating cell cycle
Source: Cell Death Dis. 2020 Oct 9;11(10):837. doi: 10.1038/s41419-020-03039-y (PMC7547105; doi:10.1038/s41419-020-03039-y)
Supplement: Supplementary file 11 — supplementary table 1-3 [file 41419_2020_3039_MOESM11_ESM.docx]

**Supplementary Table 1. Information on antibodies used in this study**

| **Antibody** | **WB** | **IHC** | **Specificity** | **Company** |
| --- | --- | --- | --- | --- |
| β-actin | 1:5000 | / | Mouse monoclonal | 14395-1-AP, Proteintech Group, China |
| SPATS2 | 1:2000 | 1:100 | Mouse monoclonal | sc-390306, Santa Cruz, USA |
| Ki-67 | / | 1:500 | Rabbit Polyclonal | Proteintech Group, China |
| CXCR4 | 1:1000 | / | Mouse monoclonal | 60042-1-Ig, Proteintech Group, China |
| MMP-2 | 1:1000 | / | Rabbit Polyclonal | 10373-1-AP, Proteintech Group, China |
| MMP-7 | 1:1000 | / | Rabbit Polyclonal | 10374-1-AP, Proteintech Group, China |
| MMP-9 | 1:5000 | / | Rabbit Polyclonal | 10375-1-AP, Proteintech Group, China |
| EZH2 | 1:1000 | / | Rabbit monoclonal | D2C9, Cell Signaling, USA |
| P21 | 1:800 | / | Rabbit Polyclonal | 10355-1-AP,Proteintech Group , China |
| P27 | 1:2000 | / | Rabbit Polyclonal | 25614-1-AP,Proteintech Group , China |
| Survivin | 1:5000 | / | Rabbit Polyclonal | 10508-1-AP,Proteintech Group , China |
| C-myc | 1:2000 | / | Rabbit Polyclonal | 10828-1-AP,Proteintech Group , China |
| Cyclin D1 | 1:5000 | / | Mouse Polyclonal | 60186-1-AP,Proteintech Group , China |
| Bcl-2 | 1:1000 | / | Rabbit Polyclonal | 12789-1-AP,Proteintech Group , China |
| Bax | 1:4000 | / | Rabbit Polyclonal | 50599-2-Ig,Proteintech Group , China |
| Bak | 1:1000 | / | Rabbit monoclonal | D4E4, Cell Signaling, USA |
| Caspase 3 | 1:5000 | / | Rabbit Polyclonal | 19677-1-AP,Proteintech Group , China |
| Cytochrome C | 1:5000 | / | Mouse monoclonal | 66264-2-Ig,Proteintech Group , China |

**Supplementary Table 2. HCC mRNA datasets obtained from GEO**

| **Cohort ID** | **platform** | **Number of samples** | | **Publication year** | **country** |
| --- | --- | --- | --- | --- | --- |
|  |  | **Non-tumor** | **tumor** |  |  |
| GSE102083 | Affymetrix | 105 | 152 | 2018 | Japan |
| GSE14520 | Affymetrix | 220 | 225 | 2003 | USA |
| GSE39791 | Illumina | 72 | 72 | 2014 | USA |
| GSE45436 | Affymetrix | 41 | 93 | 2014 | USA |
| GSE54236 | Agilent | 80 | 81 | 2014 | Italy |
| GSE57957 | Illumina | 39 | 39 | 2014 | Singapore |
| GSE60502 | Affymetrix | 18 | 18 | 2015 | Taiwan |
| GSE62232 | Affymetrix | 10 | 81 | 2014 | France |
| GSE64041 | Affymetrix | 60 | 60 | 2016 | Switzerland |
| GSE76297 | Affymetrix | 52 | 153 | 2017 | USA |
| GSE76427 | Illumina | 52 | 115 | 2017 | Singapore |
| GSE77314 | Illumina | 50 | 50 | 2016 | China |
| GSE84005 | Affymetrix | 38 | 38 | 2017 | China |
| GSE6764 | Affymetrix | 40 | 35 | 2007 | USA |
| GSE77509 | Illumina | 40 | 20 | 2017 | China |
| GSE84598 | Illumina | 44 | 22 | 2017 | Germany |

**Supplementary Table 3. HCC miRNA datasets obtained from GEO**

| **Cohort ID** | **platform** | **Number of samples** | | **Publication year** | **country** |
| --- | --- | --- | --- | --- | --- |
|  |  | **Non-tumor** | **tumor** |  |  |
| GSE6857 | OSU-CCC | 242 | 240 | 2019 | USA |
| GSE10694 | CapitalBio Mammalian | 78 | 78 | 2012 | China |
| GSE12717 | CapitalBio | 6 | 10 | 2014 | USA |
| GSE20058 | Affymetrix | 0 | 93 | 2014 | USA |
| GSE30297 | Affymetrix | 0 | 98 | 2017 | USA |
| GSE32957 | Nanostring nCounter | 8 | 27 | 2012 | USA |
| GSE36915 | Illumina | 21 | 68 | 2012 | Taiwan |
| GSE39678 | Homo sapiens 0.5K | 8 | 16 | 2012 | South Korea |
| GSE39791 | Illumina | 72 | 72 | 2016 | USA |
| GSE40744 | Affymetrix | 43 | 33 | 2012 | USA |
| GSE44570 | Affymetrix | 4 | 12 | 2017 | USA |
| GSE57957 | Illumina | 39 | 39 | 2016 | Singapore |
| GSE64989 | Affymetrix | 0 | 18 | 2015 | Germany |
| GSE67138 | Affymetrix | 0 | 57 | 2015 | USA |
| GSE67139 | Affymetrix | 0 | 120 | 2015 | USA |
| GSE74618 | Illumina | 20 | 230 | 2015 | Spain |
| GSE115016 | Affymetrix | 12 | 12 | 2018 | China |
