# Supplementary material for: SPATS2, negatively regulated by miR-145-5p, promotes hepatocellular carcinoma progression through regulating cell cycle
Source: Cell Death Dis. 2020 Oct 9;11(10):837. doi: 10.1038/s41419-020-03039-y (PMC7547105; doi:10.1038/s41419-020-03039-y)
Supplement: Supplementary file 12 — Supplementary materials and methods [file 41419_2020_3039_MOESM12_ESM.docx]

**Supplementary materials and methods**

**Cell culture and vector construction**

L02, Chang liver and HCC cell lines (HepG2, SK-Hep-3b, MHCC97-H and SMMC-7721) were obtained from the Cell Bank of the Chinese Academy of Sciences, Shanghai, China. HepG2, SK-Hep-3b and MHCC97-H cells were cultured in Dulbecco’s modified Eagle medium supplemented with 10% FBS. SMMC-7721 cells were cultured in Roswell Park Memorial Institute medium (RPMI) 1640 supplemented with 10% FBS. All cell lines were cultured at 37 °C in 5% carbon dioxide.

The GV248 (hU6-MCS-Ubiquitin-EGFP-IRES-pu-romycin) vector (GeneChem, Shanghai, China) was used to construct shRNA plasmid targeting SPATS2 (GenBank accession number: NM_178844). The shRNA sequences were listed as following: forward 5′-ccggCATTCCTGTTg-3′, and reverse 5′-CATGGCAAGTTG-3′. A scrambled sequence was used as a negative control. MiR-145 mimics and miR-145 inhibitors were purchased from GeneChem company (GeneChem, Shanghai, China).

**Immunoﬂuorescence staining**

HepG2 and SMMC-7721 cells were cultured on coverslips and fixed with 4% paraformaldehyde (w/v) for 30 minutes. Then cells were permeabilized with 0.5% Triton X-100 in PBS and blocked with BSA. After that, cells were incubated with primary anti-SPATS2 antibody at 4 °C overnight, followed by incubation with FITC conjugated secondary antibody. All slides were counterstained with 4'-6-diamidino-2-phenylindole (DAPI, Roche, Palo Alto, CA, USA). Fluorescent images were recorded and analyzed with a laser scanning confocal microscope (Olympus, Japan).

**Real-time fluorescence quantitative polymerase chain reaction (RT-qPCR)**

Total RNA was extracted by TRIzol reagent (Invitrogen, USA). RT-qPCR reactions were carried out with the ABI Prism 7500 fast sequence detection system (Applied Biosystems, USA) and DNA master SYBR Green kit (Takara Bio, Japan) according to the manufacturer's instruction. All of the reported results were the average ratios of three independent experiments. The relative mRNA expression levels were calculated using the 2^-∆∆Ct^ methods and normalized to the mRNA expression of β-actin, which was used as an internal control.

**Cell proliferation assay**

The effect of SPATS2 on cell viability was determined using Cell Counting Kit (CCK) -8 (Dojindo, Japan) assay. Briefly, HepG2 or SMMC-7721 cells were seeded in a 96-well plate (2000 cells per well) and incubated for 24 h. Then cells were transfected with different plasmids or miRNAs (sh-SPATS2/control, miR-145/miR-145&SPATS2/control), and cultured for additional 24 h, 48 h, 72 h, 96 h and 120 h, respectively. The optical density (OD) 450 nm values in each well were determined by a microplate reader. Experiments were repeated at least three times.

**EdU incorporation assay**

HepG2 or SMMC-7721 cells were seeded in a 96-well plate (2000 cells per well) and incubated for 24 h. Cells were transfected with different plasmids or miRNAs (sh-SPATS2/control, miR-145/miR-145&SPATS2/control) and then labeled with 20 μM EdU overnight. After labeling and washing, cells were fixed with formaldehyde and stained with Alexa488-azide for 20 minutes. After washing three times with PBS with 0.5% Triton X-100, cells were stained with 10 μM Hoechst for 30 minutes. Then cells were washed three times with PBS with 0.5% Triton X-100 and imaged by fluorescence microscopy.

**Cell invasion assay**

Polycarbonate membrane Transwell (Corning, USA) and Matrigel (BD, USA) were used for the invasion assay. A total of 6 × 10^4^ HepG2 or SMMC-7721 cells from different groups (transfected with different plasmids or miRNAs: sh-SPATS2/control, miR-145/miR-145&SPATS2/control) were suspended in 200 µl of serum-free medium and seeded into the upper chamber, while 600 µl of medium containing 10% FBS was added to the lower chamber. After incubation for 48 h at 37 °C, the remaining cells on the upper surface were removed with cotton swabs. The membranes were fixed in methanol and stained with 0.5% crystal violet. Cells on the lower surface of the membrane were counted in randomly selected fields with microscope (200 ×). The experiment was independently repeated three times.

**Colony formation assay**

Long-term survival of cells was assessed by the ability of colony formation. Briefly, HepG2 or SMMC-7721 cells from different groups (transfected with different plasmids or miRNAs: sh-SPATS2/control, miR-145/miR-145&SPATS2/control) were seeded at 500 cells/well in culture dishes. After 14 days, clones were counted following being ﬁxed with 75% ethanol and stained with 0.2% crystal violet (Sigma, St. Louis, MO, USA). All experiments were repeated three times.

**Luciferase reporter assay**

Luciferase reporter vector with the full length of the 3’-UTR of SPATS2 were constructed. Then we generated the mutant luciferase reporter vectors with QIAGEN XL-site directed Mutagenesis Kit (QIAGEN, USA). HepG2 cells were seeded into 96-well plates and co-transfected with luciferase reporter vector and miR-145 mimics using the Lipofectamine 2000 transfection reagent. After 48 h of incubation, the firefly and Renilla luciferase activities were quantified with a dual-luciferase reporter assay (Promega, USA).

**RNA immunoprecipitation assay**

RIP assays were performed to validate the direct binding between miR-145 and SPATS2 according to the manufacturer’s instructions (Magna RIP RNA-Binding Protein Immunoprecipitation Kit. Millipore, USA). Briefly, HepG2 cells were lysed by the NP-40 lysis buffer and then incubated with RIP buffer containing magnetic beads bound with human anti-Argonaute2 (Ago2) antibody (Millipore) or normal mouse IgG (Millipore) as a negative control. Precipitate was digested with Proteinase K buffer, and then the complexes of RNA were then treated with Trizol (Life Technologies) for further purification and qRT-PCR.
